# Adaptive markers distinguish North and South Pacific Albacore amid low population differentiation

**DOI:** 10.1111/eva.13202

**Published:** 2021-02-23

**Authors:** Felix Vaux, Sandra Bohn, John R. Hyde, Kathleen G. O'Malley

**Affiliations:** ^1^ State Fisheries Genomics Lab Coastal Oregon Marine Experiment Station Department of Fisheries and Wildlife Hatfield Marine Science Center Oregon State University Newport OR USA; ^2^ Department of Zoology University of Otago Dunedin New Zealand; ^3^ Southwest Fisheries Science Center National Marine Fisheries Service La Jolla CA USA

**Keywords:** adaptation, genome, highly migratory species, population genetic, RADseq, seascape genomics

## Abstract

Albacore (*Thunnus alalunga*) support an economically valuable global fishery, but surprisingly little is known about the population structure of this highly migratory species. Physical tagging data suggest that Albacore from the North and South Pacific Ocean are separate stocks, but results from previous genetic studies did not support this two stock hypothesis. In addition, observed biological differences among juveniles suggest that there may be population substructure in the North Pacific. We used double‐digest restriction site‐associated DNA sequencing to assess population structure among 308 Albacore caught in 12 sample areas across the Pacific Ocean (10 North, 2 South). Since Albacore are highly migratory and spawning areas are unknown, sample groups were not assumed to be equivalent to populations and the genetic data were analyzed iteratively. We tested for putatively adaptive differences among groups and for genetic variation associated with sex. Results indicated that Albacore in the North and South Pacific can be distinguished using 84 putatively adaptive loci, but not using the remaining 12,788 presumed neutral sites. However, two individuals likely represent F1 hybrids between the North and South Pacific populations, and 43 Albacore potentially exhibit lower degrees of mixed ancestry. In addition, four or five cross‐hemisphere migrants were potentially identified. No genetic evidence was found for population substructure within the North Pacific, and no loci appeared to distinguish males from females. Potential functions for the putatively adaptive loci were identified, but an annotated Albacore genome is required for further exploration. Future research should try to locate spawning areas so that life history, demography, and genetic population structure can be linked and spatiotemporal patterns can be investigated.

## 1 INTRODUCTION

Population structure is challenging to assess in highly migratory marine species (HMMS), which hinders the development of effective management and conservation strategies. From a practical standpoint, it is hard to comprehensively sample HMMS year‐round across their vast geographic ranges, which typically span the open ocean and extend across numerous international boundaries. As a result, genetic sampling is often temporally and spatially limited, and relies on collaborations across fishery sectors. These sampling challenges often lead to infrequent records of age, sex, and length, which means that ontogenetic, demographic, and phenotypic patterns associated with the genetic samples may not be detected. In addition, the location and timing of reproduction are often unknown for HMMS (e.g., Whale Sharks, Schmidt et al., 2009), making it difficult to assign individuals back to discrete spawning areas compared with anadromous, freshwater, or terrestrial taxa.

Marine species typically have large effective population sizes (*N*_e_), panmictic populations with frequent dispersal and high gene flow, and low genetic variation (Nielsen et al., 2009; Waples et al., 2008). However, this general pattern is not always true, and differences in life history and oceanography can result in population structure within HMMS. For example, the Blue Marlin *Makaira nigricans* (Lacépède, 1802) and the Sailfish *Istiophorus platypterus* (Shaw, 1792) are ecologically similar HMMS, except that movements of the Sailfish appear to be restricted to coastal waters (Ortiz et al., 2003). Genetic data from these species indicate that Blue Marlin from the Pacific and Indian oceans represent a single panmictic population (Chen, Chang, et al., 2016), whereas Sailfish in the North Pacific Ocean are readily distinguished into separate eastern and western populations (Lu et al., 2015).

Previous population genetic studies of HMMS have mostly used microsatellites, and although some have detected low genetic differentiation indicative of panmixia (e.g., Chen, Chang, et al., 2016; Schmidt et al., 2009; Veríssimo et al., 2017), others have identified substantial differences between populations from different oceans (e.g., Muths et al., 2013). Microsatellites typically capture only neutral genetic variation and do not reflect adaptive differences among groups. In contrast, recent SNP‐based studies have identified more subtle population structure based on variation at putatively adaptive loci. For example, a restriction site‐associated DNA sequencing (RADseq) analysis of Striped Marlin *Kajikia audax* (Philippi, 1887) revealed six temporally consistent populations, using 59 putatively adaptive loci (Mamoozadeh et al., 2020). These results significantly differed from previous genetic studies that identified four populations, which are currently recognized as stocks for fisheries management (McDowell & Graves, 2008; Purcell & Edmands, 2011).

In this study, we focused on the highly migratory Albacore *Thunnus alalunga* (Bonnaterre, 1788). Albacore are fast, efficient swimmers capable of traveling over 110 km per day (Childers et al., 2011). The species is small compared with most tunas (Collette & Nauen, 1983), but individuals may reach ~140 cm in fork length and 60 kg in body weight (Evano & Bourjea, 2012; Otsu & Sumida, 1970). Albacore are circumglobal and occur predominantly between 40°N and 40°S (Collette & Nauen, 1983; Erauskin‐Extramiana et al., 2019; Nikolic et al., 2017); however, their exact geographic range is uncertain in some regions (Nikolic et al., 2017). Prevailing migration cycles have been hypothesized in the Atlantic and Pacific oceans but spawning areas are only broadly estimated to large regions between 10 and 25°N/S, and the distribution and movement of young juveniles (<2 years old) is unclear (ISSF, 2019; Nikolic et al., 2017). Individuals typically live 15–21 years (Wells et al., 2013), and reproductive maturity occurs between 5 and 6 years old (Nikolic et al., 2017; Otsu & Sumida, 1970). The oldest age classes (at FL >95–100 cm) exhibit a male‐biased sex ratio, which is likely caused by female‐biased mortality (Farley et al., 2013).

Albacore support a large, global fishery (ISC, 2017; ISSF, 2019) and are the fourth most‐landed tuna species globally (FAO, 2014) and an important economic resource for many small and developing states (Dhurmeea et al., 2016). Stocks of Albacore are currently managed under four Regional Fisheries Management Organizations (RFMOs) (Nikolic et al., 2017). Six stocks aligned with ocean basins are currently recognized (i.e., North Atlantic, South Atlantic, Indian, North Pacific, and South Pacific oceans, and the Mediterranean Sea; Davies et al., 2011; Nikolic et al., 2017). None of these stocks are considered overfished, but catches of Albacore are highly variable and there is considerable uncertainty for some estimates (ISC, 2017; ISSF, 2019). The boundary between the northern and southern stocks in the Atlantic and Pacific oceans is between 5°N and 0°N and 5°N and 5°S, respectively (Nikolic et al., 2017). Tagging studies have mostly supported these boundaries (Ichinokawa et al., 2008; Nikolic et al., 2017), although occasional migrations have been recorded between the North Atlantic and Mediterranean (Arrizabalaga et al., 2002, 2003; Ortiz de Zárate et al., 2011). Catch rates in equatorial waters between the North and South Pacific are also very low (Nikolic et al., 2017).

Most population genetic research on Albacore has used microsatellites, short‐range PCR products, or tens to hundreds of presumed neutral SNPs, and sampling has varied considerably (Table S1). In concordance with the currently recognized stocks and tagging data, Albacore from Atlantic Ocean, Pacific Ocean, and the Mediterranean Sea were consistently distinguished from one another (e.g., Nakadate et al., 2005; Viñas et al., 2004). However, a potential east‐west genetic cline in the Mediterranean remains uncertain (Davies et al., 2011; Laconcha et al., 2015; Montes et al., 2012). The genetic distinction of Albacore in the Indian Ocean has been challenging. A recent study was able to distinguish samples from the southwest Atlantic and southwest Indian oceans (Nikolic et al., 2020), but earlier research detected a high level of admixture (Davies et al., 2011; Laconcha et al., 2015; Montes et al., 2012), suggesting that there may be frequent migration from the Atlantic and Pacific oceans into the Indian Ocean.

**FIGURE 1:**
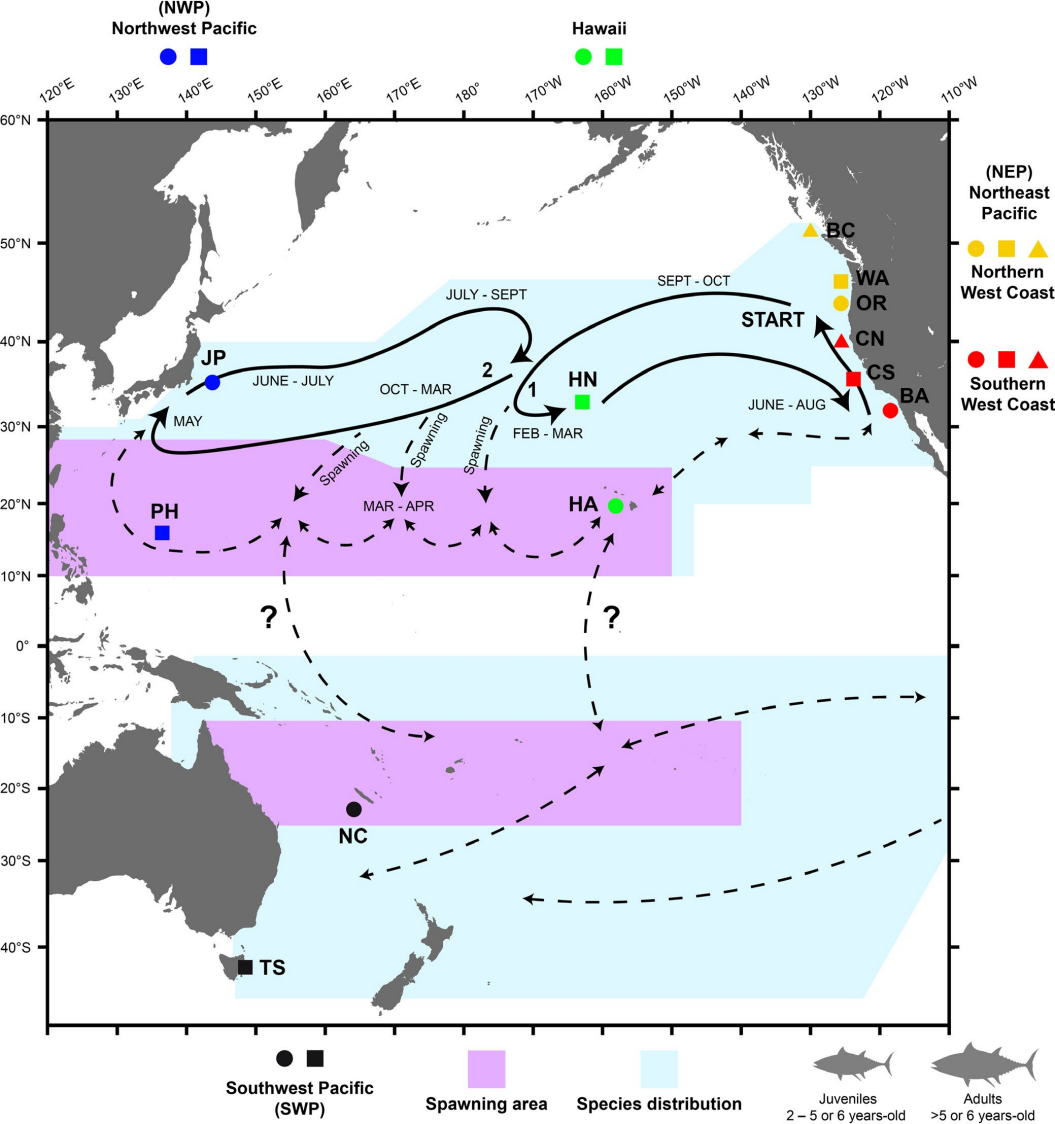
A Mercator projection map of the Pacific Ocean showing the distribution of Albacore, the hypothesized migration cycle, and the sample areas for this study. Following previous research (Nikolic et al., 2017; Otsu & Uchida, 1963), blue shading indicates distribution of Albacore, and purple areas estimate the approximate range of spawning areas. The 12 sample areas are labeled with unique symbols and two letter abbreviations (listed in Table 1). The sample area symbols are colored by region: northeast Pacific (NEP; red for southern and yellow for northern West Coast), Hawaii (green), Northwest Pacific (NWP; blue), and Southwest Pacific (SWP; black). The general migratory pattern is that juveniles (2–5 or 6 years old) occur in the central and northeast North Pacific Ocean. Both juveniles and adults leave Northeast Pacific waters in September/October and migrate west (“Start”), arriving at a region centered on 40°N, 170°E in February/March. Juveniles can migrate in two directions at this point. Younger fish (<3 years old) tend to migrate eastward and reach waters off California and Baja California by June–August (“1”). Older juveniles (>3 years old) tend to continue migrating westward, where they reach southern Japan by May, and then subsequently leave Japan in June/July before migrating back to the 40°N, 170°E region (“2”). As fish age, they increasingly migrate westward toward Japan. Adults are hypothesized to migrate southward from the 40°N, 170°E region (“Spawning”), arriving at the equatorial spawning area in March/April. Many adults appear to rejoin the migration cycle, still favoring migration toward Japan, but after spawning, some adults potentially remain below 30°N or adopt an alternative migration cycle (dashed lines)

Population structure in the North Pacific is of particular interest as the region contains one of the largest Albacore fishing stocks (ISC, 2017; ISSF, 2019), and because migratory behavior in this region indicates potential population substructure (Nikolic et al., 2017). Based on tagging data, Otsu and Uchida (1963) hypothesized a migratory cycle for North Pacific Albacore that has remained largely unmodified (Ichinokawa et al., 2008; Kimura et al., 1997; Nikolic et al., 2017; Figure 1). The general migratory pattern is that young juveniles (<3 years) are predominantly found in the Northeast Pacific, and they migrate toward a region centered on 40°N, 170°E in February/March, before returning by June/August (Figure 1). However, older juveniles (3–5 or 6 years old) increasingly migrate westward toward Japan as they age, forming two ellipses across the North Pacific (Figure 1). Adults are believed to migrate southward from the 40°N, 170°E region to spawn in equatorial waters in March/April (Figure 1). Many adults appear to rejoin the migration cycle, but some adults potentially remain below 30°N or migrate elsewhere (Figure 1).

Evidence from juvenile Albacore indicates that there may be population substructure within the northeast Pacific Ocean. Some juveniles within the southern range overwinter west of Baja California (see dashed line on Figure 1), rather than migrating northward during late summer (Childers et al., 2011). In addition, juveniles sampled above and below 40°N along the west coast of North America differ in size and growth rate (Childers et al., 2011; Laurs & Lynn, 1977; Nikolic et al., 2017; Renck et al., 2014). The stable isotope composition of muscle tissue differs between Albacore from these regions as well, and, although otolith cores are indistinguishable, the isotopic signatures of otolith edges are significantly different, which indicates that juveniles occupy regions with similar isotopic signatures but move into different waters as they age (Wells et al., 2015).

Recently, a RADseq study of Albacore in the Southwest Pacific analyzed thousands of SNPs that were separated into putatively adaptive and presumed neutral loci (Anderson et al., 2019). As shown in previous studies, there was no evidence for population substructure within the Southwest Pacific based on the neutral loci (Anderson et al., 2019). However, one area was distinguished from three others using 89 putatively adaptive loci, but it was unclear whether this distinction was due to spatial or temporal differences (Anderson et al., 2019). RADseq studies that sampled widely across the range of Yellowfin Tuna *Thunnus albacares* Bonnaterre, 1788 have also identified discrete population structure using putatively adaptive loci (Grewe et al., 2015; Pecoraro et al., 2018), despite previous research using neutral markers failing to identify such structure (e.g., Díaz‐Jaimes & Uribe‐Alcocer, 2006; Ward et al., 1994).

We investigated population genetic structure among Albacore sampled in the Pacific Ocean, and determined whether fish sampled in the North and South Pacific could be distinguished from one another. Genetic variation was analyzed using double‐digest restriction site‐associated DNA sequencing (ddRADseq; Peterson et al., 2012), and we tested for presumably neutral and putatively adaptive differences among potential populations. Like many studies of HMMS (e.g., Bernard et al., 2016; Brendtro et al., 2008; Grewe et al., 2015; Mamoozadeh et al., 2020), and most genetic studies of albacore (e.g., Albaina et al., 2013; Anderson et al., 2019), we were not able to link samples to separate spawning areas. Given this situation, we explicitly acknowledged that our sample areas do not necessarily represent separate populations, and we iteratively analyzed the ddRADseq data to avoid any a priori biases. In addition, since sex biases can lead to inaccurate interpretations of population genetic structure (Benestan et al., 2017; Catchen, 2017), we also investigated whether the likely male‐biased, adult sample groups of Albacore are separated in the population genetic data. Furthermore, we tested for genetic differentiation between the sexes, as shared loci can differ between males and females if there is intralocus sexual conflict (e.g., Vaux et al., 2019), and tested whether loci aligned with the sex‐determining regions of the Pacific Bluefin genome (Suda et al., 2019).

## 2 METHODS

### 2.1 Sampling

A total of 361 Albacore were sampled from 12 sample areas in the Pacific Ocean (Table 1, Figure 1, Appendix S1). All fish were caught by commercial fishing or research vessels using troll, hook‐and‐line bait fishing, and commercial longline usually over several consecutive days, within an area <50 km^2^. Most fish were lethally sampled, except for individuals caught off Northern California and Washington that were released alive as part of an archival tagging study (Childers et al., 2011; Appendix S1). Fish from each sample area were collected across different years and seasons between 2005 and 2018 (Table 1). All samples were collated by National Marine Fisheries Service (NMFS) or by the Pacific Community (SPC) Pacific Marine Specimen Tissue Bank (https://www.spc.int/ofp/PacificSpecimenBank).

**TABLE 1:** Sampling of North Pacific Albacore for ddRADseq by sample area

Pacific Ocean region	West Coast subregion	Label on Figure 1	Sample area	Collection period	# Successfully sequenced samples	Average length and standard deviation (cm)	Age class	Note
NEP	Southern	BA	Baja California, Mexico	Sep 2006	4	80.7 (7.9)	Juvenile	
NEP	Southern	CS	Southern California, USA	Dec 2012	39	71.0 (14.3)	Juvenile	
NEP	Southern	CN	Northern California, USA	Oct 2010	19	79.8 (3.5)	Juvenile	†
NEP	Northern	OR	Oregon, USA	Sep 2009	31	77.0 (4.1)	Juvenile	*
NEP	Northern	WA	Washington, USA	Oct 2013	27	74.3 (5.4)	Juvenile	†
NEP	Northern	BC	British Columbia, Canada	Oct 2005	10		Juvenile	
Hawaii		HA	Hawaii, USA	Nov–Dec 2016	25	114.2 (7.7)	Adult	
Hawaii		HN	Northwest of Hawaii	Jun 2010	28	94.8 (8.9)	Adult	
NWP		JP	Shimizu, Japan	Feb 2016	22	73.1 (7.6)	Juvenile	
NWP		PH	Philippine Sea	Dec 2012	29	103.1 (5.9)	Adult	
SWP		NC	New Caledonia, France	May–Sept 2014	54	92.0 (5.9)	Adult	36 M, 18 F
SWP		TS	Tasmania, Australia	Mar 2013 Mar 2018	20		Juvenile	
				**Total**	**308**			

Note

All length measurements are fork length (FL), except for samples from Oregon that used total length (TL), which is marked with an asterisk (*). Length measurements were not available for samples from British Columbia and Tasmania. Samples from New Caledonia were sex‐identified, with 36 males and 18 females. See Appendix S1 for further information on an individual basis, including tissue type and decimal coordinates for sampling locations.

**FIGURE 2:**
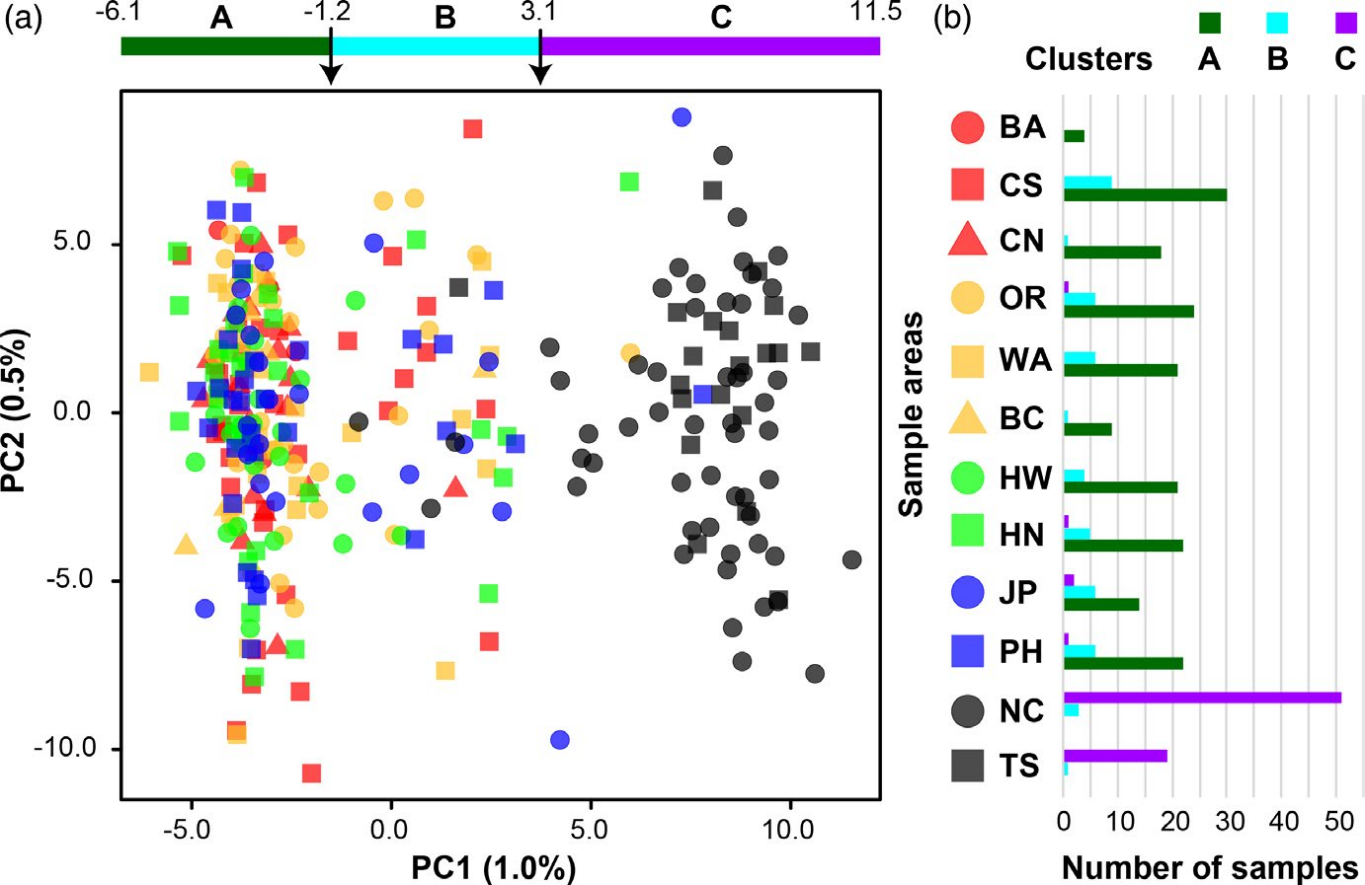
Principal components analysis (PCA) results for the reference mapped 12 sample area dataset (6446 loci). (a) A scatterplot presenting genetic variation among all samples, with samples classified by sample area. A bar with arrows above the plot denotes the potential separation of individuals into three clusters (A–C) along PC1 (1.0% of SNP variation). (b) A histogram showing membership of the three clusters (A–C) identified in the PCA scatterplot

The shading of rows matches colors used for sample groups used in Figures 1, 2, 4 and 5.

Fork length (FL) or total length (TL) was recorded in cm for all samples, except for fish from British Columbia and Tasmania that were not measured (Table 1, Appendix S1). Based on length measurements and previous distribution studies (Table 1, Figure 1; Nikolic et al., 2017), the sample areas likely represent a mixture of juveniles (2–5 or 6 years old) and adults (>5 or 6 years old). For example, Albacore caught off Northern California are likely juveniles (mean FL = 79.8 cm, SD 3.5 cm), whereas fish from Hawaii (mean FL = 114.2 cm, SD 7.7 cm) are well above the minimum size of maturity (~85 cm; Chen, Hsu, et al., 2016). Sex was recorded for fish from New Caledonia, with 36 males and 18 females sampled (Table 1). Sex was unknown for the remaining samples, although the Hawaii and Philippine Sea sample areas with many fish above 95–100 cm FL (Table 1) were noted as being likely to exhibit a male‐biased sex ratio (Otsu & Sumida, 1970; Otsu & Uchida, 1959; Xu et al., 2016).

### 2.2 DNA extraction and ddRADseq

A tissue sample (fin, muscle, or liver) was taken from each individual. Tissue samples were transferred to separate 1.5‐ml microtubes and preserved in either 95% ethanol or RNAlater (Sigma‐Aldrich). All sample processing was conducted at Oregon State University's Center for Genome Research and Biocomputing.

Genomic DNA was isolated from ~50 mg of tissue using a DNeasy Blood and Tissue Kit (Qiagen) and normalized to 20 ng/µl. Each sample was double‐digested in a 20 µl reaction using the high fidelity restriction enzymes *PstI*‐*HF* (6 bp cutter, 5′…CTGCAG… 3′) and *SphI*‐*HF* (6 bp cutter, 5′…GCATGC… 3′) (New England Biolabs, Inc.) at 37°C for 2 h. Following digestion, 91 barcoded adapters and a common adapter were ligated to individual samples using T4 Ligase (New England Biolabs) at 22°C for 2 h, and heat‐inactivated at 65°C for 30 min. All samples were pooled and cleaned using QIAquick PCR Purification Kit (Qiagen) and 200‐ to 500‐bp fragments were isolated on a Blue Pippin (Sage Science). Size‐selected fragments were then amplified with Illumina primers under the following conditions: 98°C 30”; 98°C 10”, 68°C 30”, 72°C 30” (15 cycles); and 72°C 5’, 4°C hold. The PCR product was purified, eluted, and quantified using a Qubit fluorometer (Thermo Fisher Scientific). The final library consisted of 361 Albacore organized onto four 96‐well sequencing plates, with 91 or 92 individuals per plate, sequenced on separate lanes. The four library plates were each run on an Illumina HiSeq 3000 lane using 150‐bp paired‐end sequencing chemistry.

### 2.3 Processing of ddRADseq data

A total of 1,546,541,006 DNA read pairs were sequenced (Table S2). stacks 2.41 (Rochette et al., 2019) was used to process reads, identify loci, and estimate genotypic variation. See Figure S1 for a summary of the analytical pipeline. Forward and reverse reads from each index were demultiplexed into separate inline barcodes using the *process_radtags* component of the stacks pipeline. The *process_radtags* step removed reads with low‐quality read data or with ambiguous barcodes and RAD tags, which resulted in a total of 1,383,807,804 read pairs being retained (Table S2). This step included the rescue barcode and RADtag parameter (‐r) to retrieve additional reads.

### 2.4 Reference map assembly of loci and filtering

Paired sequence reads were mapped to reference genomes using the BWA‐MEM algorithm in bwa 0.7.12 (Li & Durbin, 2009). Three tuna reference genomes were investigated: a contig assembly of Pacific Bluefin (RefSeq: GCA_000418415.1; Nakamura et al., 2013), a scaffold assembly of Atlantic Bluefin (RefSeq: GCA_003231725.1; Puncher et al., 2018), and a scaffold of Yellowfin (RefSeq: GCA_900302625.1). The mean percentage of successfully mapped paired reads was estimated by the flagstats command in samtools 1.4 (Li et al., 2009) and used to determine the most suitable reference genome (Table S3). The Pacific Bluefin was selected as a reference genome, with the highest mean mapping success at 93.9%.

Loci and genotypes were called for mapped reads using the *ref_map* pipeline in stacks. Default settings were applied, with variant sites and genotypes called at a significance level of 5% in *gstacks* (‐‐var_alpha 0.05 ‐‐gt‐alpha 0.05). Population genetic variation was estimated using the *populations* component of the stacks pipeline. Following the recommendation of Mastretta‐Yanes et al. (2015), we modified parameters in *populations* to examine the ddRADseq data comprehensively (Table S4). As recommended by Fountain et al. (2016), we investigated the relationship between coverage depth per locus and an estimated heterozygote miscall rate for each variation of *population* parameters, using the whoa 0.01 R package (Anderson, 2018; R Core Team, 2019). We selected final *populations* settings (and datasets) based on this error rate, the number of variant loci, and a conservative aim to avoid a high number of potentially erroneous reads.

Three datasets were selected for investigation. In the “12 sample area dataset,” all individuals were organized into the 12 sample areas (‐p 12). In the “North and South Pacific dataset,” all individuals were organized into North and South Pacific groups (‐p 2). In the “New Caledonia sex dataset,” all 54 Albacore from New Caledonia were organized into two groups for males and females (‐p 2). The minimum percentage of individuals in a population required to process a locus for a given population was set at either 90% or 95% (‐r 0.9 or 0.95). A minimum allele frequency of 5% was enforced for loci (‐‐min_maf 0.05). Only the first SNP of each locus was included (‐‐write_single_snp), and all SNPs were biallelic.

For each dataset, we tested the conformance of loci to the Hardy–Weinberg proportions (HWP) in each group (e.g., 12 sample areas; Table 2) using vcftools 0.1.16 (Danecek et al., 2011). In the 12 sample area dataset, loci that did not conform to HWP in at least four populations were recorded. HWP estimation used an exact test (Wigginton et al., 2005), and we corrected for multiple tests using a false discovery rate (FDR) adjustment for *p*‐values with a critical threshold of <5% (Allendorf et al., 2013; Bouaziz et al., 2012; Storey, 2002; Waples, 2015).

**TABLE 2:** Settings applied in the *populations* component of stacks 2.41 for the three reference mapped datasets, and number of loci filtered and retained for analysis

*n*	Dataset	Locus representation		# HWP	Filtering					# Final variant and invariant loci	# Final variant loci (SNPs)	Final mean heterozygote miscall rate
		# pops	Populations		# PSVs	# LD	# Genotype depth <10	# Loci depth outliers	Total excluded loci			
308	12 sample areas	12	‐p 12 ‐r 0.90	0	95	5	92	215	402	16,434	6446	4.7%
308	North and South Pacific	2	‐p 2 ‐r 0.95	0	183	17	787	519	1490	31,523	12,872	4.3%
54	New Caledonia sex	2	‐p 2 ‐r 0.95	0	62	9	1785	451	2300	33,786	6	3.6%

Note

The table lists how many loci were estimated and removed for being paralogous sequence variants (PSVs), for evidence of linkage disequilibrium (LD), for having low coverage depth, or for being coverage depth outliers. All loci conformed to the Hardy–Weinberg proportions (HWP) in all tested groups in each of the three datasets after the correction for multiple tests. Subsequently, no loci were removed for HWP. The last column provides the estimated heterozygote miscall rate for each dataset after filtering.

Dark grey shading helps to emphasise the important numbers of loci to focus on for each dataset.

Loci in all three datasets were filtered to mitigate potential biases (e.g., coverage depth effects, sequencing artifacts) in the raw RAD sequencing data that can produce misleading genotypic results (Fountain et al., 2016; O'Leary et al., 2018). Putatively paralogous sequence variants (PSVs) were identified using the python and R scripts for hdplot (McKinney et al., 2017) and the modified script paralog‐finder 1.0 that accounts for varying degrees of missing data per locus among individuals (Mortiz, 2018). Loci estimated to be in linkage disequilibrium (LD) were identified using plink 1.9 with a cutoff of 0.8 (Purcell et al., 2007). In the 12 sample area and New Caledonia sex datasets, all samples were placed into one group to estimate LD, whereas in the North and South Pacific dataset, samples were organized into the two Pacific Ocean groups. Loci with individual genotypes below a coverage depth of 10 reads (‐‐minDP 10) and with ≥20% missing data among all individuals (‐‐max‐missing 0.8) were identified using vcftools 0.1.16. The low coverage depth loci, putative PSVs, and one locus from each pair estimated to be in LD were organized into a list and excluded (‐B; Catchen et al., 2013), and the *populations* component of stacks was rerun (same settings as above) so that these sites were removed from subsequent analyses (Table 2). Variation in coverage depth per locus was investigated in the subsequent dataset using the vcfr R package (Knaus & Grünwald, 2017), and loci that were outliers for mean coverage depth and the standard deviation of coverage depth were identified (Table 2). These coverage depth outlier loci were added to an updated, second list of excluded loci and the *populations* component of stacks was rerun for a final time (Table 2, Figure S1). Coverage depth per individual and per locus, and the heterozygote miscall rate were estimated for all datasets after filtering to gauge filtering success (Table 2). The format of output files from stacks was converted for analyses in downstream software using pgdspider 2.1.1.3 (Lischer & Excoffier, 2012).

### 2.5 De novo assembly of loci

Since reads for Albacore were mapped to the reference genome of a different species, we assembled the demultiplexed ddRADseq reads using the *de_novo* pipeline in stacks. Methods and results for this analysis, which did not significantly differ from the reference mapped analyses, are presented in Appendix S2.

Following the recommendation of previous research (Gouin et al., 2015; Laine et al., 2019), we also attempted a de novo assembly of the reads (~6%) that did not successfully map to the Pacific Bluefin reference genome. However, only a handful of variant loci could be recovered from these reads while maintaining a low heterozygote miscall rate (Table S4). It was therefore concluded that unmapped reads represented an insignificant proportion of genetic variation, which was not investigated further.

### 2.6 Genetic variation

After filtering loci, genetic variation was investigated for the 12 sample area, North and South Pacific Ocean, and New Caledonia sex datasets. Observed (*H*_O_) and expected heterozygosity (*H*_E_), allelic richness, and the inbreeding coefficient (*F*_IS_) were estimated for each tested group (i.e., sample area, ocean, or sex) using the adegenet 2.1.1 R package (Jombart, 2008; Jombart & Ahmed, 2011). The level of relatedness among individuals in each sample area and in the North and South Pacific groups, in the corresponding datasets (‐p 12 and 2), was assessed using the Wang relatedness estimator implemented in coancestry 1.0.1.9 (Wang, 2011). The Wang relatedness estimator is appropriate for small sample sizes (<50 individuals) with many loci (Wang, 2017).

Genetic variation among individuals and the tested groups was explored using principal components analysis (PCA), again implemented in adegenet (Jombart, 2008; Jombart & Ahmed, 2011). All loci within each dataset were used for PCA. We determined the number of “meaningful” principal components (PCs) to retain for interpretation and downstream analyses by comparing PC eigenvalues.

### 2.7 Detection of putatively adaptive loci

Putatively adaptive loci were estimated independently using four genome scan programs: fsthet 1.01 (Flanagan & Jones, 2017), outflank 0.2 (Whitlock & Lotterhos, 2015), bayescan 2.1 (Foll & Gaggiotti, 2008), and pcadapt 4.0.3 (Luu et al., 2016). Four separate programs were used to identify putatively adaptive loci, as the stringency of loci classification, false discovery rate, and the fit of applied models to particular patterns of genetic variation are known to vary among methods (see discussion, Ahrens et al., 2018; Flanagan & Jones, 2017; Gagnaire et al., 2015; Luu et al., 2016; Narum & Hess, 2011). Marine populations with large effective population sizes and wide geographic distributions can also be particularly challenging for the reliable identification of putatively adaptive loci (Gagnaire & Gaggiotti, 2016), which warrants the use of multiple programs. Differences between these four programs are described in Vaux et al. (2019).

Default settings were used for fsthet and outflank (Flanagan & Jones, 2017; Whitlock & Lotterhos, 2015). In bayescan, we used default parameters and a prior of 100, with a *q*‐value threshold of 0.05 (analogous to an FDR of 5%; Foll & Gaggiotti, 2008), and output data were investigated using the boa 1.1‐8‐2 R package (Smith, 2007). In pcadapt, we applied the default settings, including an alpha value of 0.1, and only the first two PCs were analyzed for each dataset. The qvalue 2.12.0 R package was used to estimate FDR for pcadapt (Storey et al., 2018). Given the underlying assumptions of pcadapt (Luu et al., 2016), outlier detection results from the program were treated as negative (no outlier loci) if there was no obvious population structure for any PC, and if all PCs had low eigenvalues. As a conservative precaution against Type I error, only loci identified as outliers by at least three programs were organized into putatively adaptive datasets and separated from the remaining, presumed neutral loci. Without information from future annotated genome or selection studies, we interpret all loci as only putatively adaptive and presumed neutral (Gagnaire & Gaggiotti, 2016; Nielsen et al., 2009; Shafer et al., 2015). Without such information, it is not possible to exclude the potential influence of genetic incompatibilities on observed allelic frequencies (Bierne et al., 2011). Similarly, allele surfing—where alleles at the leading edge of a range expansion can sharply increase in frequency—could also be confused with a pattern of genetic adaptation (Excoffier & Ray, 2008).

### 2.8 Genetic differentiation and hybrid detection

Genetic differentiation was examined among the groups in the three datasets. Weir and Cockerham's (1984) pairwise fixation index (*F*_ST_) was estimated for groups using putatively adaptive loci, presumed neutral loci, and all loci in each dataset. This index was estimated using the stampp 1.5.1 R package (Pembleton et al., 2013), with 5000 bootstraps and an FDR adjustment for *p*‐values with a critical threshold of <5% for the *F*_ST_ *p*‐values, using the same method as described for HWP estimation.

Discriminant analysis of principal components (DAPC) in adegenet was used to visualize the differentiation between groups in the North and South Pacific datasets, using three sets of loci: putatively adaptive loci, presumed neutral loci, and all loci. Each set of loci tested with DAPC used 1 discriminant function, and 20 PCs were used for all loci and the presumed neutral loci, whereas only 1 PC was used for the putatively adaptive loci.

Genetic population structure was investigated among groups in the 12 sample area and North and South Pacific datasets using Bayesian genotypic clustering in structure 2.3.4 (Pritchard et al., 2000). We tested for up to five potential genotypic clusters among individuals (*K* = 1–5). For each value of *K*, five replications of the admixture model with independent allele frequencies were applied, with an MCMC length of 50,000 generations and a 10% burn‐in. The optimal number of clusters was determined by examining estimates of mean *K* probability for a given value of *K* (Pritchard et al., 2000) and deltaK, the rate of change in logarithmic probability of the data (Evanno et al., 2005) implemented in structure harvester 0.6.94 (Earl & vonHoldt, 2012).

The presence of individuals with northern and southern population mixed ancestry, hereafter referred to as “hybrids,” was investigated by comparing individual cluster assignment credibility values in structure. In addition, first‐generation (F1) hybrid individuals were estimated in geneclass 2.0h (Piry et al., 2004), using the frequency‐based, simulation method of Paetkau et al. (2004) with 1000 simulated individuals, a critical *p*‐value threshold of 0.01.

### 2.9 Identity of putatively adaptive loci

Since the Pacific Bluefin genome assembly is comprised of unannotated contigs, the identity of the putatively adaptive Albacore loci could not be determined using reference mapping alone. The Pacific Bluefin contigs containing putatively adaptive Albacore loci were therefore aligned with DNA sequences available on the NCBI GenBank database using NCBI blastn (Altschul et al., 1990). Alignment success was determined using alignment score, E value, and shared % identity. Gene annotations and human Entrez IDs were recorded for sequences that successfully aligned to the contigs. These human Entrez IDs were analyzed in panther 14.1 (Mi et al., 2019) against the human genome to estimate potential biological functions (Panther GO‐slim biological processes) for these regions in the Albacore. Although the Albacore and human genomes are highly divergent, such distant species comparisons can still identify numerous conserved vertebrate gene functions (Boffelli et al., 2004). A statistical overrepresentation test using the Bonferroni correction was implemented in panther to test whether any biological processes occurred more often than expected by chance.

To further assess the identity of the putatively adaptive loci, we also compared our genetic results with a previous RADseq study of Albacore (Anderson et al., 2019). We trimmed adapter sequences from their loci sequences and mapped them to the Pacific Bluefin genome contigs using the same BWA‐MEM method. We then compared the position of those markers with the position of our putatively adaptive loci. We note that the two RADseq datasets are unlikely to include the exact same loci due to differences in the size selection and sequencing read length. Our mapping method may also fail to detect many nearby loci as the Pacific Bluefin contigs are short (length range 428–79,059 bp). Lastly, we tested whether any putatively adaptive loci detected in this study mapped to the sex‐determining regions identified in a recently sequenced Pacific Bluefin genome (GCA_009176245.1; Suda et al., 2019).

## 3 RESULTS

### 3.1 Sequencing and loci

A total of 53/361 individuals failed to successfully sequence, resulting in a final sample of 308 Albacore (Table 1; Appendix S1). Using all 308 successfully sequenced individuals, the 12 sample area dataset had 6446 loci, whereas the North and South Pacific dataset had 12,872 loci (Table 2). The smaller dataset comparing only the 36 males and 18 females from New Caledonia had 6917 loci (Table 2). After controlling for multiple testing, no loci across all datasets showed significant deviation from the Hardy–Weinberg proportions (Table 2).

After filtering, locus coverage depth per individual was fairly consistent across all sample areas, although samples from Baja California and British Columbia had lower depths. This difference likely reflects the poor quality of tissue for these sample areas, where the majority of the 47 specimens failed to successfully sequence. As an example, Figure S2 presents mean coverage depth per individual in the 12 sample area dataset. After filtering, coverage per locus was well constrained to a near‐normal distribution with no obvious outliers; example plots are shown for the 12 sample area dataset in Figure S3. Filtering resulted in a low heterozygote miscall rate (<5%) for every dataset (Table 2 and Table S5, Figure S4).

### 3.2 Genetic variation for each dataset

#### 3.2.1 12 sample areas

In the 12 sample area dataset (6446 loci), slightly fewer heterozygotes were observed than expected for all of the sample areas, except Baja California (Table 3). This slight deficiency in heterozygotes was reflected with positive *F*_IS_ values (Table 3). Results for Baja California (*n* = 4) and British Columbia (*n* = 10) were likely influenced by the limited sample sizes for these areas. Hawaii exhibited a lower *F*_IS_ value relative to NW of Hawaii and other sample areas (Table 3). However, given that samples from Hawaii and NW of Hawaii were collected quite far apart (hundreds of km) and 6 year apart (Figure 1; Table 1), there is no inherent reason to assume that these sample groups would have matching summary statistics. A deficiency in heterozygotes in sample areas could have been caused by genotyping error, but analysis in whoa estimated that the heterozygote miscall rate was low (4.7%) (Table 2, Figure S4). Alternatively, a deficiency in heterozygotes could have been caused by sampling a higher number of related individuals than expected by chance, but according to the Wang relatedness estimator, there was no evidence of high relatedness (>0.25) within and among the 12 sample areas (Table S6). These results therefore suggest samples from Hawaii were simply slightly more diverse relative to other areas. Allelic richness was also similar among the 12 sample areas (Table 3).

**TABLE 3:** Genetic summary statistics for the reference mapped 12 sample area and North and South Pacific Ocean datasets

Map label	Group	*n*	Mean *H*_O_	Mean *H*_E_	Mean (CI) *F*_IS_	Mean AR
**12 sample area dataset**						
BA	Baja California	4*	0.17	0.17	−0.019 (−0.029 to 0.003)	1.51
CS	Southern California	39	0.17	0.18	0.048 (0.047–0.055)	1.54
CN	Northern California	19	0.17	0.18	0.035 (0.034–0.524)	1.54
OR	Oregon	31	0.17	0.18	0.042 (0.041–0.052)	1.55
WA	Washington	27	0.17	0.18	0.047 (0.045–0.061)	1.54
BC	British Columbia	10*	0.18	0.18	0.004 (−0.007 to 0.012)	1.54
HW	Hawaii	25	0.16	0.17	0.022 (0.018–0.028)	1.51
HN	NW of Hawaii	28	0.17	0.18	0.043 (0.042–0.055)	1.54
JP	Japan	22	0.18	0.18	0.042 (0.039–0.060)	1.55
PH	Philippine Sea	29	0.17	0.18	0.046 (0.044–0.058)	1.53
NC	New Caledonia	54	0.17	0.18	0.035 (0.029–0.036)	1.53
TS	Tasmania	20	0.18	0.18	0.038 (0.037–0.055)	1.55
**North and South Pacific dataset**						
	North Pacific	234	0.19	0.20	0.045 (0.038–0.047)	1.99
	South Pacific	74	0.20	0.20	0.033 (0.025–0.035)	1.99

Note

Estimated values are presented for observed (*H*_O_) and expected heterozygosity (*H*_E_), the inbreeding coefficient (*F*_IS_), and allelic richness (AR). 95% confidence intervals are presented for *F*_IS_ values. The asterisk (*) for Baja California and British Columbia indicates that these estimates are less reliable to due to limited sample sizes.

The shading of rows matches colors used for sample groups used in Figures 1, 2, 4 and 5.

Using PCA, only the first PC (1% of SNP variation) was meaningful based on PC eigenvalues (Figure S5a). The 10 sample areas from the North Pacific were separated from the two South Pacific sample areas using PC1, but no sample areas within the North or South Pacific could be distinguished (Figure 2a). However, three clusters (A–C) were apparent along PC1 (Figure 2a). Cluster A contained most samples from the North Pacific, Cluster C contained most samples from the South Pacific, and Cluster B (*n* = 48) contained a mixture of North and South Pacific individuals (Figure 2a,b). Membership of Cluster B did not appear to be significantly biased toward any of the 12 sample areas (Figure 2b). Notably, five individuals from the North Pacific were similar to samples from the South Pacific and within the range of Cluster C (Figure 2a). These individuals were one juvenile from Oregon, two juveniles from Japan, one adult from Northwest of Hawaii, and one adult from the Philippine Sea.

**FIGURE 3:**
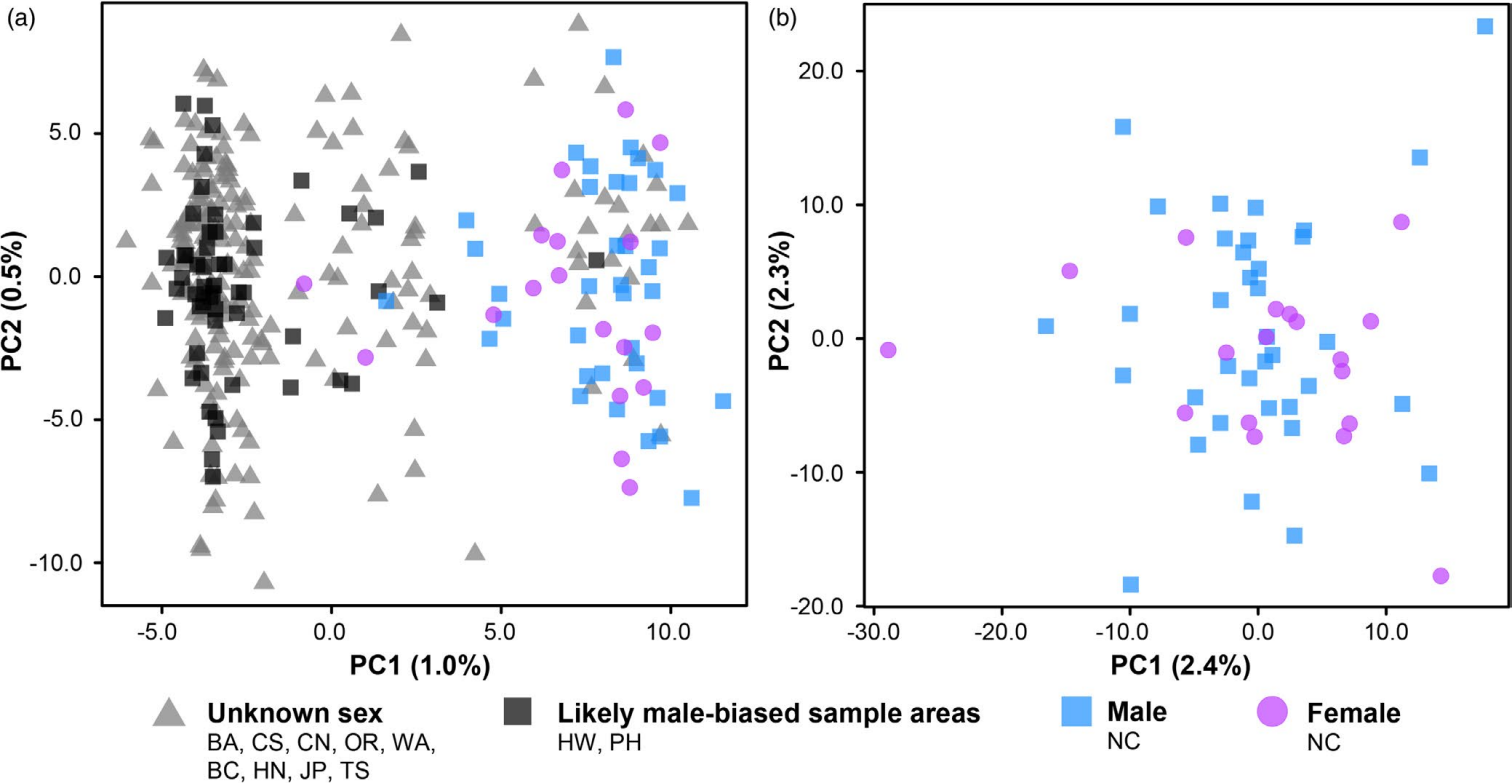
Principal components analysis (PCA) results for genetic variation associated with sex. In both plots, samples from New Caledonia were sex‐identified and are therefore classified as males or females (blue squares and purple circles, respectively). (a) A scatterplot of genetic variation among samples in the 12 sample area dataset (6446 loci), also presented in Figure 2a, but with samples classified by sex. The sample areas of Hawaii and the Philippine Sea were likely to be male‐biased and are therefore classified separately (black squares), and all remaining samples are classified as unknown (gray triangles). (b) A scatterplot of genetic variation among samples in the New Caledonia sex dataset (6917 loci)

Alternative patterns of genetic structure were explored within the 12 sample area dataset. Collection year did not reveal any clear pattern among samples (Figure S6a). Likewise, categorizing individuals by age class (juveniles or adults) did not show any patterns (Figure S6b). Sex also did not appear to be influential in the 12 sample area dataset, as PCA did not separate the 36 males and 18 females from New Caledonia, or distinguish the likely male‐biased sample areas of Hawaii and the Philippine Sea (Figure 3a). In addition, none of these sample areas exhibited unusual biases for summary statistics (Table 3).

#### 3.2.2 North and South Pacific

The North and South Pacific dataset exhibited similar patterns of genetic variation to the 12 sample area dataset, but it contained almost twice as many loci (12,872 vs. 6446; Table 2). This increase most likely reflected the interaction of stacks parameters and group size. There were slightly fewer heterozygotes observed than expected for the North and South Pacific groups, which was reflected by positive *F*_IS_ values (Table 3). The *F*_IS_ value was lower for the South Pacific, which may reflect the lower sample size for this group (*n* = 74). Genotyping error was again considered to be unlikely to have caused this result, as analysis in whoa estimated a low heterozygote miscall rate (4.3%) for the North and South Pacific dataset (Table 2, Figure S4). Likewise, the Wang estimator found no evidence of high relatedness (≥0.25) within and between the North and South Pacific groups (Table S7), and so it is unlikely that the sampling of a high number of related individuals could have generated the slight deficiency in heterozygosity. The allelic richness of the two populations was also similar (Table 3).

**FIGURE 4:**
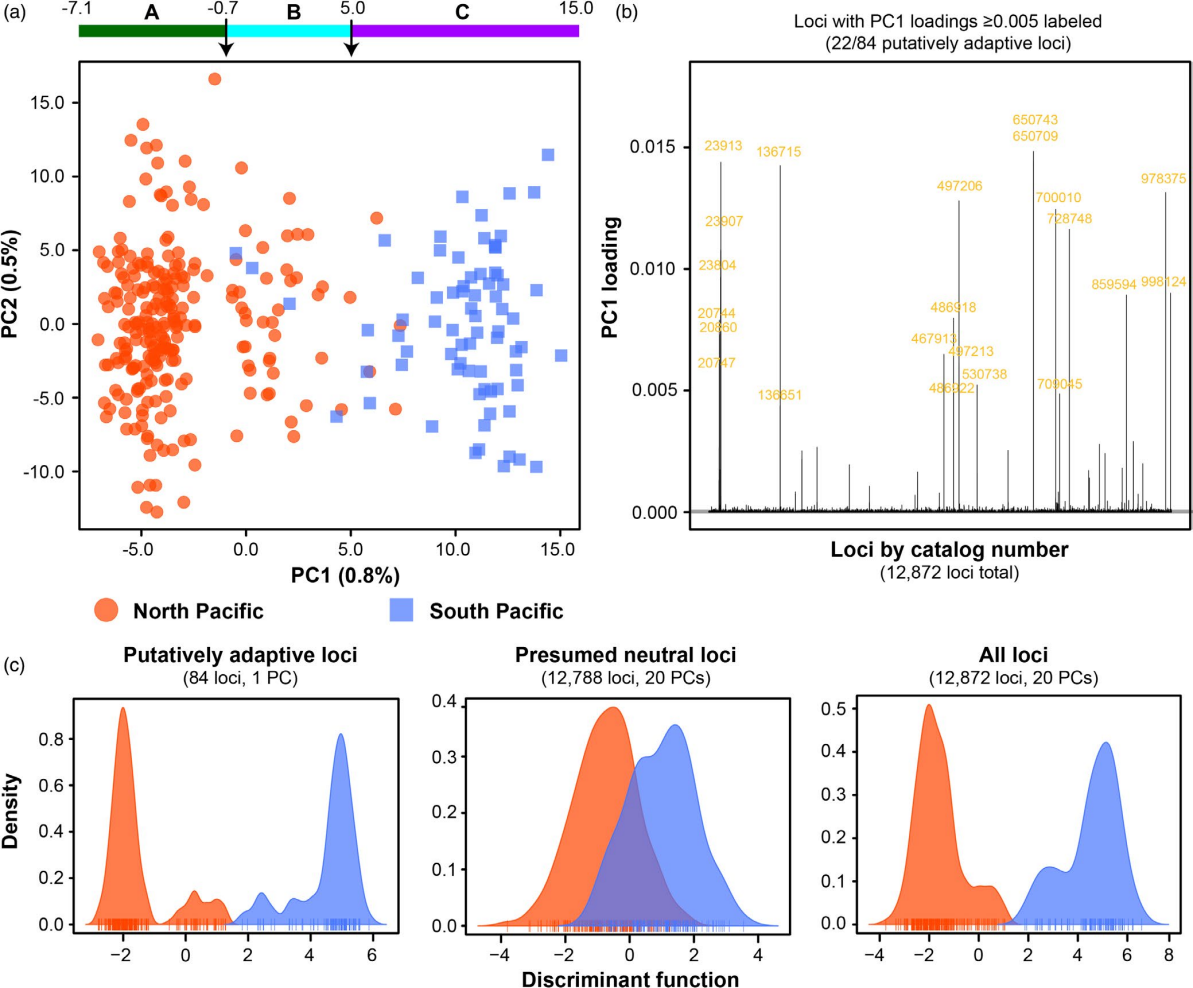
Genetic variation in the North and South Pacific Ocean dataset (total of 12,872 loci) based on principal components analysis (PCA) and discriminant analysis of principal components (DAPC). (a) A PCA scatterplot of genetic variation among samples, but with samples classified by sampling origin within the North or South Pacific. (b) Loadings for all loci for PC1 (0.8% of variation) in the PCA. Almost all loci with visible loadings in this plot were identified as putatively adaptive loci. Loci with PC1 loadings >0.005 are labeled in yellow, which represent 22 of the 84 identified putatively adaptive loci. (c) DAPC plots for the putatively adaptive loci (84 loci, using 1 PC), the presumed neutral loci (12,788 loci, using 20 PCs), and all loci (12,872 loci, using 20 PCs). All DAPCs used 1 discriminant function, comparing the North and South Pacific populations

Using PCA on the North and South Pacific dataset, PC1 (1.0%) was again the only meaningful axis of variation based on eigenvalues (Figure S5b). As with the 12 sample area dataset, three clusters could be distinguished along PC1 (Figure 4a). The composition of these clusters was similar, with Cluster B (*n* = 45) containing a mixture of North and South Pacific individuals (Figure S7). Again, no other groups could be distinguished using PCA. Four Albacore sampled in the North Pacific exhibited South Pacific genotypes, with one juvenile each from Washington, British Columbia, and Japan, and one adult from Hawaii (Figure 4a and Figure S7). These four individuals did not overlap with the five identified in the 12 sample area dataset.

#### 3.2.3 New Caledonia sex

Sex was investigated again more specifically using only the sex‐identified individuals in the New Caledonia sex dataset (6917 loci). Based on analysis in whoa, the estimated heterozygote miscall rate for the dataset was low (3.6%) (Table 2, Figure S4). According to eigenvalues, no PC was meaningful (Figure S5c), but the first two PCs for this dataset (collectively 4.7% of variation) were examined anyway, and males and females could not be distinguished (Figure 3b).

### 3.3 Detection of putatively adaptive loci in the North and South Pacific dataset

Since the only prevailing genetic pattern across all three loci datasets was the separation between the North and South Pacific, we focused on the North and South Pacific dataset for subsequent analyses. However, some of these downstream analyses were replicated for the 12 sample area dataset in order to check for consistency in results, which are presented in Appendix S2. Results between datasets were highly congruent.

Putatively adaptive loci were identified by all four genome scan programs in the North and South Pacific dataset (Table 4). Putatively adaptive loci were organized into a separate list if they were identified by at least three programs, and the remaining loci were categorized as presumed neutral sites. Overall, a final set of 84 putatively adaptive loci were identified (Table 4 and Table S11). Putatively adaptive loci identified by each of the four genome scan programs are provided in Appendix S3.

**TABLE 4:** Putatively adaptive loci estimated for each reference mapped dataset using the four genome scan programs: fsthet, outflank, bayescan, and pcadapt

Dataset	*n*	# Groups	Total # loci	# Putatively adaptive loci estimated				Final datasets	
				fsthet	OutFLANK	BayeScan	pcadapt	# Putatively adaptive	# Presumed neutral
North and South Pacific	308	2	12,872	371	126	72	92	84	12,788

Dark grey shading used to emphasise that these are the final numbers of loci used for the putatively adaptive and presumed neutral loci datasets.

As expected, given the lack of alternative genetic structure, there was a strong relationship between the putatively adaptive loci and PC1, which distinguished samples from the North and South Pacific. This relationship could be observed using the PC1 loadings for loci, where loci with the largest loadings were all identified as putatively adaptive sites (Figure 4b). As recommended to assess the accuracy of the fsthet analysis (Flanagan & Jones, 2017), comparisons of *F*_ST_ and observed heterozygosity (*H*_O_) for loci are shown in Figure S9.

### 3.4 Genetic differentiation and hybrid detection

Using only the 84 putatively adaptive loci, the pairwise *F*_ST_ value between the North and South Pacific populations was high at 0.4055, whereas the *F*_ST_ estimate for the remaining presumed neutral loci was low at 0.0005 (Table 5). Using all 12,872 loci, the pairwise *F*_ST_ value was also low at 0.0056, but statistically significant (Table 5). This pattern reveals that the small number of putatively adaptive loci contributed substantially to the overall estimate of genetic differentiation.

**TABLE 5:** Pairwise *F*_ST_ values for the putatively adaptive, presumed neutral, and all loci in the North and South Pacific dataset. Pairwise *F*_ST_ values for the 12 sample areas are presented in Figure S10

# Loci	Pairwise *F*_ST_ (95% CI) *p*‐value
Putatively adaptive loci 84	0.4055 (0.3313–0.4727)
Presumed neutral loci 12,788	0.0005 (0.0003–0.0006) <0.0001
All loci 12,872	0.0056 (0.0039–0.0075) <0.0001

North and South Pacific samples were distinguished by DAPC using the putatively adaptive loci (1 PC), presumed neutral loci (20 PCs), and all loci (using 20 PCs; Figure 4c). The two groups exhibited a greater degree of overlap using only presumed neutral loci. For the DAPC of the putatively adaptive loci and all loci, a subset of individuals were not as easily distinguished as others (Figure 4c), which reflected the occurrence of Cluster B, as observed in the PCA plot (Figure 4a).

**FIGURE 5:**
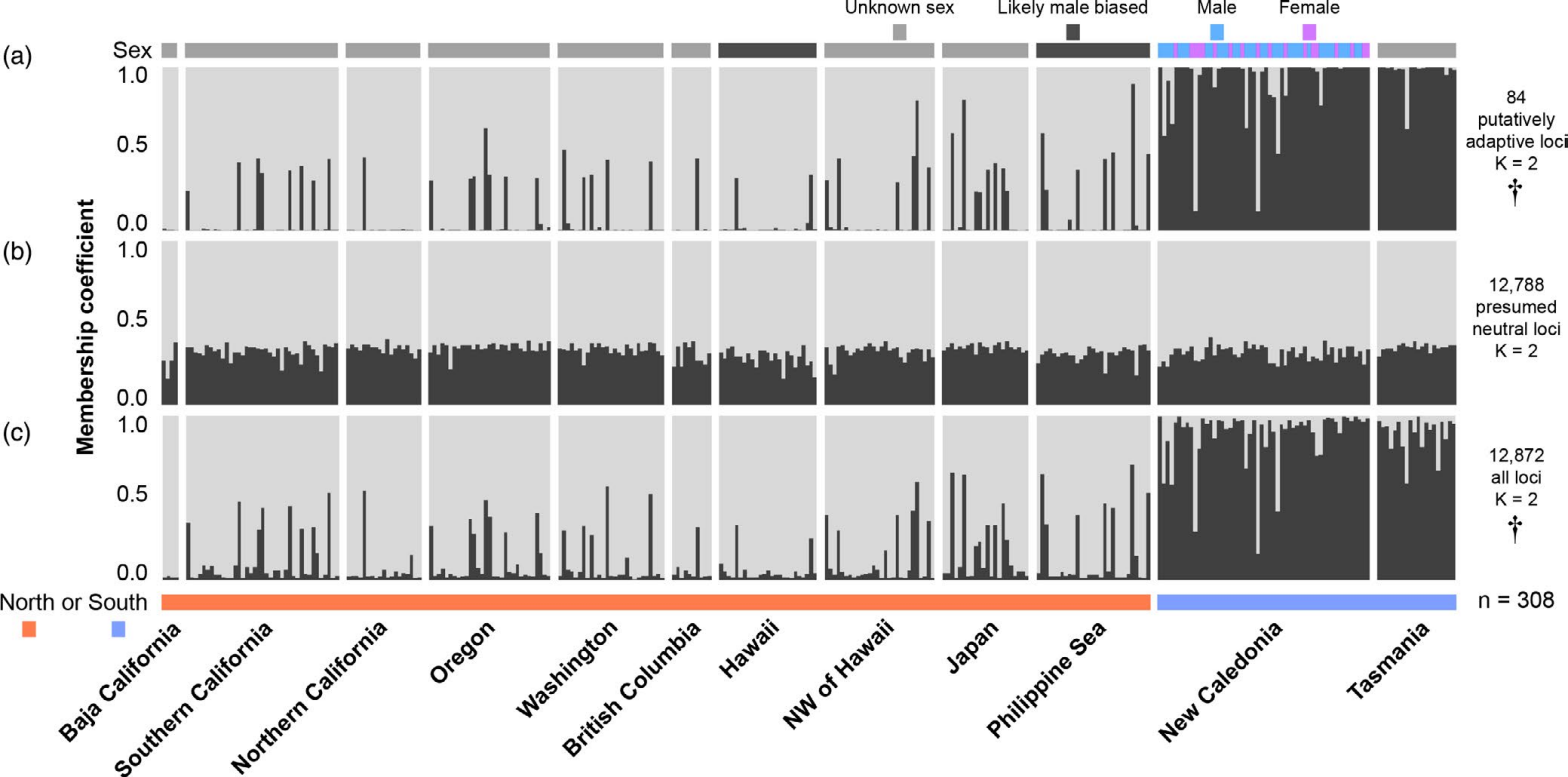
structure bar graphs showing genotypic clusters estimated among samples in the North and South Pacific Ocean dataset. Bar graphs show results for (a) the putatively adaptive loci, (b) the presumed neutral loci, and (c) all loci. Within a graph, each vertical bar represents a separate individual and the genotypic clusters estimated among individuals are shown in different colors (light gray and charcoal). The height of a cluster within each vertical bar indicates the confidence that a particular individual was assigned to a given genotypic cluster (referred to as the membership coefficient). A bar above the three graphs denotes the sex of samples from New Caledonia, and for the remainder of samples, it specifies whether sex is unknown or whether the sample area (Hawaii and the Philippine Sea) is likely to be male‐biased. The colored bars below the graphs indicate North or South Pacific origin, and the 12 sample areas are labeled. The dagger sign (†) indicates whether the presented clustering model was the optimal number of clusters for each set of loci. The optimal clustering model for presumed neutral loci (b) is not shown, as it was one cluster

The genotypic clusters estimated by structure supported similar patterns to the PCA, pairwise *F*_ST_, and DAPC results. In the North and South Pacific dataset, two clusters were identified as being optimal for the putatively adaptive loci, one cluster was optimal for the presumed neutral loci, and two clusters were optimal for all loci (Figure S10). The two clusters identified for the putatively adaptive loci and all loci clearly distinguished most Albacore from the North and South Pacific (Figure 5a,c). No individuals were distinguished for neutral loci even under a suboptimal two‐cluster model (Figure 5b). As with the PCA, males and females and the three likely male‐biased sample areas of Hawaii and the Philippine Sea were not distinguished by the structure analysis (Figure 5).

In the structure analysis of putatively adaptive loci, some individuals (*n* = 35) exhibited near‐even assignment (0.4–0.6) between the two clusters. Of these individuals, seven were assigned to Cluster B identified in the PCA plot (Figure 4a). In addition, three out of the four North Pacific individuals that were similar to samples from the South Pacific in PCA plots (in Cluster C) were clustered with South Pacific samples. The remaining fourth PCA individual had even assignment between the clusters identified by structure. It is possible that these 35 individuals with near‐even assignment are of mixed ancestry between the potential North and South Pacific populations. To investigate this, we examined the 95% credibility intervals for the cluster assignment of individuals in the North and South Pacific dataset using the 84 putatively adaptive loci (Figure S13). Most of the 35 individuals with near‐even cluster assignment had constrained 95% credibility intervals, indicating that the structure analysis was confident of their mixed identity.

We tested whether any of the 45 Cluster B individuals (identified in the PCA) could be identified as F1 hybrids using the 84 putatively adaptive loci in geneclass2. Results indicated that 5/45 Cluster B individuals were identified as F1 hybrids, along with four additional individuals from the North Pacific (Table S13). Furthermore, four individuals identified as F1 hybrids by geneclass2 exhibited near‐even cluster assignment (0.4–0.6) in structure (Table S13 ). No other individuals with near‐even assignment in structure were identified as F1 hybrids by geneclass2. Overall, one individual from the Philippine Sea and another from New Caledonia were identified as potential hybrids by all three methods (Table S13).

### 3.5 Identity of putatively adaptive loci

The 84 putatively adaptive loci in Albacore mapped to 74 Pacific Bluefin genome contigs (Table S11). The contigs were between 1372 and 38,856 bp in length (mean 11,188 bp). Using blastn, 65 out of 74 of these contigs successfully aligned with fish DNA sequences on GenBank. Of these, 37 contigs aligned with genes, six aligned with microsatellites, and the remaining 22 sequences matched with unannotated contigs for other fish genome sequencing projects (Table S14). Of the 37 gene matches, 31 were unique (some contigs aligned to the same genes; Table S14). Eleven loci directly aligned to genes, but the remaining 64 loci with gene matches aligned adjacent to genes within a contig (Table S14). Analysis in panther identified potential biological functions for 20 out of the 31 genes, meaning that 26 of the 84 putatively adaptive loci in Albacore could be traced to a wide range of potential biological functions in humans (Table S15). However, after the Bonferroni correction for multiple tests, no particular potential biological function occurred more often than expected by chance.

We compared our 84 putatively adaptive loci with the 89 putatively adaptive loci identified by Anderson et al. (2019) among Albacore in the southwest Pacific Ocean. Using bwa, four pairs of loci between the two datasets mapped to the same contigs (Table S16). One pair of loci were directly adjacent, sharing the same *PstI* restriction site, but using different *SphI* restriction sites. Based on blastn alignment of the Pacific Bluefin contigs, these four regions represented two genes: “interferon regulatory factor 5” and “ubiquitin carboxyl‐terminal hydrolase 28‐like,” and two microsatellites applied in previous fish studies (Table S14). In addition, none of the putatively adaptive loci mapped to the sex‐determining regions identified in Pacific Bluefin (Suda et al., 2019).

## 4 DISCUSSION

### 4.1 Genetic differentiation between North and South Pacific populations

Most of the Albacore samples from the North and South Pacific could be readily distinguished using a small subset of putatively adaptive loci. However, the majority of presumed neutral loci suggest a pattern of panmixia across the Pacific Ocean. These patterns were apparent using PCA, DAPC, genotypic clustering in structure, and in the pairwise *F*_ST_ results for both the 12 sample area and the North and South Pacific datasets (Table 5, Figures 2, 4, and 5, Figure S8). Such a difference in resolution between putatively adaptive and presumed neutral loci has been reported by previous RADseq studies of tuna (Grewe et al., 2015; Pecoraro et al., 2018), and other studies of marine fishes (Longo et al., 2020; Mamoozadeh et al., 2020). Overall, our results demonstrate the power of putatively adaptive loci to identify population structure in HMMS (Gagnaire et al., 2015; Gagnaire & Gaggiotti, 2016).

### 4.2 Evidence of migration and hybridization

Although populations in the North and South Pacific could be distinguished from one another, results suggest that migration and gene flow occur across the equator. Three genetic clusters (A–C) were apparent in the PCA plots using both the 12 sample area and North and South Pacific datasets (Figures 2 and 4). Clusters A and C corresponded to the majority of samples from the North and South Pacific, respectively, whereas Cluster B represented a smaller number of individuals with apparently mixed genetic origin. In the North and South Pacific dataset, structure analysis had high confidence that 35 samples exhibited mixed North and South Pacific genetic identity, and seven of these individuals belonged to Cluster B in the PCA plot (Figure S12). Furthermore, analysis in geneclass2 identified some of these individuals as potential F1 hybrids between the North and South Pacific populations (Table S13). Different sets of individuals were identified as potential hybrids by PCA, structure, and geneclass2, which likely reflects the statistical differences among these methods and the difficulty of reliably detecting hybrid individuals in general (Anderson, 2008). However, one adult from the Philippine Sea and another from New Caledonia were identified as hybrids by all three methods (Table S13). Taking a conservative approach, we suggest that at least two sampled Albacore are F1 hybrids and 43 further individuals potentially exhibit lower degrees of mixed ancestry between the North and South Pacific.

Results for PCA also indicated that some Albacore sampled in the North Pacific had South Pacific genotypes. In the 12 sample area dataset, five individuals sampled in the North Pacific exhibited Cluster C genotypes, with three juveniles from Oregon and Japan, and two adults from Northwest of Hawaii and the Philippine Sea (Figure 2). In the North and South Pacific dataset, four separate individuals were instead identified, with three juveniles from Washington, British Columbia, and Japan, and one adult from Hawaii (Figure 4 and Figure S7). Using the 84 putatively adaptive loci in the North and South Pacific dataset, all five Cluster C individuals in the 12 sample area dataset were identified as F1 hybrids by geneclass2 (Table S13). In contrast, none of the four Cluster C individuals in the North and South Pacific dataset were identified as F1 hybrids. According to structure analysis of the North and South Pacific dataset, four of these nine individuals were assigned to the South Pacific cluster, three had mixed genotypes with constrained 95% credibility values, and two were assigned to the North Pacific cluster. Altogether, these results suggest that some of these individuals could be migrants from the South Pacific to North Pacific, but the distinction between F1 hybrids and potential migrants is difficult (Anderson, 2008) and clearly affected by the groups used for the identification of loci and downstream population genetic analyses. Furthermore, since the five and four Cluster C individuals sampled in the North Pacific for the 12 sample area and North and South Pacific datasets did not overlap, the support for these migrant individuals is weaker than the consistent identification of the two potential F1 hybrids. For these results, we also stress that genetic and demographic populations are not necessarily equivalent, for instance, if mating is nonrandom (Sugg et al., 1996). Ideally, future estimates of gene flow should be complemented with direct measures of migration (e.g., physical tagging) in order to accurately estimate population connectivity (Lowe & Allendorf, 2010; Waples et al., 2008).

Altogether, our genetic results for Albacore differ from past tagging studies that did not observe cross‐hemisphere migration in the Pacific Ocean (Childers et al., 2011; Ichinokawa et al., 2008; Otsu, 1960; Otsu & Uchida, 1963). However, these studies only sampled juveniles, which may exhibit different migratory behavior compared with adults. As hypothesized by Nikolic et al. (2017), adults could migrate between the spawning areas on each side of the equator, but frequent tagging would be required to capture such movement if it is relatively rare. Catch per unit effort data indicate that Albacore are rare within equatorial waters (Nikolic et al., 2017), but this does not necessarily mean that cross‐hemisphere migration is uncommon. Albacore fishing gear targets surface waters, but the species can forage below 500 m (Nikolic et al., 2017), and it is possible that spawning adults exhibit behavior or foraging preferences that significantly reduce catch rates at the equator.

A total of 46 juvenile Albacore from Northern California and Washington sequenced in this study were live sampled and tagged by Childers et al. (2011). These juveniles were assigned to different PCA clusters in the North and South Pacific dataset: Cluster A (38 samples), B (seven samples), and C (one sample), which indicates a mixture of genetic profiles (Appendix S1). Unfortunately, none of these fish had successful tag returns, and so it is not possible to compare this genetic variation with recorded migratory behavior.

### 4.3 No evidence of population substructure within the North Pacific

Using both putatively adaptive and presumed neutral loci, we did not identify population structure to support the distinction of separate stocks in the northeast Pacific Ocean. Northern and southern West Coast groups were previously identified among juveniles based on body size and growth rate (Laurs & Lynn, 1977; Renck et al., 2014), and otolith isotope composition (Wells et al., 2015). Tagging research also indicates that some juveniles within the southern West Coast region overwinter west of Baja California, rather than migrating northward (Childers et al., 2011). Our genetic results suggest that these northern and southern groups do not represent distinct genetic populations. The most likely explanation for this pattern is that young Albacore reside and feed in different areas, resulting in phenotypic differences, but with age, they no longer remain in separate areas. It is possible that sampling over multiple seasons and years could identify subtle genetic differences between these groups. After all, it can be difficult to genetically differentiate closely related populations if mixed stocks occur (Waples et al., 2008). Fine‐scale population structure in marine species with large population sizes is also likely to result in very low *F*_ST_ values, which can make very fine population structure difficult to distinguish from a false negative result (Gagnaire et al., 2015; Lowe & Allendorf, 2010). However, given that we sampled 130 Albacore along the West Coast from six sample areas and thoroughly analyzed thousands of loci, it is unlikely that any potential genetic differentiation among sample areas in the North Pacific is comparable to the strong differentiation observed between the North and South Pacific using the putatively adaptive loci.

The lack of genetic population structure in the North Pacific is congruent with the only previous genetic study of Albacore that sampled multiple areas in the North Pacific, which could not distinguish Albacore sampled off Japan, Taiwan, and Hawaii using mitochondrial DNA sequence data (Wu et al., 2009). A recent RADseq study of Yellowfin Tuna also did not detect population substructure within the North Pacific (Pecoraro et al., 2018), albeit with more limited spatial sampling.

### 4.4 No evidence for genetic influence of sex, temporal variation, or age class

Sex did not appear to influence genetic results (Figures 3 and 5). Similarly, PCA did not distinguish New Caledonia or the likely male‐biased sample areas of Hawaii and the Philippine Sea from the other sample areas (Figure 2a). In addition, no putatively adaptive loci were identified between the sexes (Table 4), and none of the 84 putatively adaptive loci in the North and South Pacific dataset mapped to the sex‐determining regions recently identified in Pacific Bluefin (Suda et al., 2019). These results may seem surprising compared with recent RADseq results for other fishes (e.g., Benestan et al., 2017; Carrier et al., 2020; Vaux et al., 2019), but there is no guarantee for RADseq data to capture sex‐linked genetic variation. Restriction sites are typically unevenly distributed across a genome (Lowry et al., 2017), meaning that few reads may have been recovered from sex‐determining regions in the Albacore genome, and if rare, these sequences may have been lost during reference mapping, loci calling, or filtering. An improved reference genome for Albacore that includes sex‐determining regions, or a different set of restriction enzymes, could allow for sex‐linked loci to be recovered. Our results suggest that sex is unlikely to be a confounding source of genetic variation for analyses of population structure in Albacore, including a previous study (Anderson et al. 2019). Future research should investigate whether sex is influential in the genetic analysis of other tuna species, and whether it has affected past RADseq studies for Atlantic Bluefin (Puncher et al., 2018) and Yellowfin (Grewe et al., 2015; Mullins et al., 2018; Pecoraro et al., 2018). Further research needs to determine whether the sex‐determining regions identified in Pacific Bluefin (Suda et al., 2019) occur in other tunas.

Based on PCA, no patterns were apparent based on collection year or age class (Figure S6). This result suggests that genetic variation among Albacore in the Pacific Ocean has not changed considerably between 2006 and 2018, or at least that such variation is minimal compared with the spatial variation between samples from the North and South Pacific. This finding is concordant with the results of Anderson et al. (2019), in which Albacore sampled at two different time points from New Caledonian waters were more similar to each other than other geographic sample areas. Alternatively, our analysis may have lacked the power to detect temporal differences in population structure due to small sample size and irregular geographic and temporal sampling over the 12‐year period.

### 4.5 Identity of putatively adaptive loci

The 84 putatively adaptive loci mapped to 74 Pacific Bluefin genome contigs, and 65 of these contigs successfully aligned to fish DNA sequences on GenBank (Tables S11 and S14). These sequence matches included 31 unique genes and six microsatellites. Notably, four contigs containing a total of six loci aligned to “heat shock protein beta‐1” (AB438031.1) sequenced in Pacific Bluefin (Ojima & Oohara, 2009). Analysis of the 31 aligned genes in panther indicated that a broad range of potential biological functions in humans for most genes (e.g., cellular processes and metabolism and signaling; Table S14), although no biological functions were estimated to be statistically overrepresented compared to chance. Heat shock protein beta‐1 was categorized as a system process by panther, meaning a multicellular process conducted by tissue or organs, and it is known to be expressed in response to a wide range of biotic and abiotic stressors in fish (Iwama et al., 1998; Ojima & Oohara, 2009). Overall, 26 of the 84 putatively adaptive loci in Albacore were traced to biological functions that may be subject to selection. Although most estimated biological functions were often restricted to the cellular level, it is possible that they could be related to the biological differences observed between North and South Pacific Albacore. A recently sequenced scaffold genome assembly for Pacific Bluefin (Suda et al., 2019) may improve the identification of some loci, but ultimately an annotated genome assembly for Albacore is required for more accurate insight towards the potential function of the putatively adaptive loci in this particular species. A chromosome‐level genome assembly for albacore would also allow researchers to determine whether any genomic regions are enriched for putatively adaptive loci, which could be caused by chromosomal inversions or similar processes.

Using a similar DNA sequencing method, Anderson et al. (2019) identified 89 putatively adaptive loci that distinguished Albacore sampled off French Polynesia from other sample areas in the southwest Pacific Ocean. Our putatively adaptive loci mapped to four regions in common with the Anderson et al. putatively adaptive loci: “interferon regulatory factor 5” and “ubiquitin carboxyl‐terminal hydrolase 28‐like,” and two microsatellites applied in previous fish studies (Table S13). These results suggest that at least part of the genetic differentiation identified by Anderson et al. (2019) reflects the North and South Pacific pattern observed in our study. Given that French Polynesia was the most northern sample area in that study, it is possible that these individuals experience similar selective pressures as Albacore sampled in the North Pacific or that a significant number of migrant adults from the North Pacific were included among these fish.

### 4.6 Implications for Albacore fisheries management and conservation

#### 4.6.1 Two Pacific Ocean stocks amid gene flow

Although the overall results of this study support the current two stock management approach of Albacore in the Pacific Ocean (ISC, 2017; ISSF, 2019; Nikolic et al., 2017), future management and conservation efforts should consider the impact of cross‐hemisphere migration and potential rate of gene flow. For example, do migration rates vary through time, and do Albacore from certain regions migrate across the equator more often than others? It is possible that fish harvests within certain areas within the Pacific Ocean could be catching a mixture of North and South Pacific Albacore. If so, the frequency of such overlap could affect the management of both stocks. The degree of mixing among populations is important for stock delimitation and fisheries management (Hawkins et al., 2016), and the risk of introgression between distinct populations is a concern for the management of conservation units in other species (Bohling, 2016; Cairns et al., 2017; Horreo et al., 2011; Taylor et al., 2020). A similar pattern of population structure amid gene flow may be observed for other HMMS, and the same management concerns likely apply to ongoing research focused on tuna, billfish, and sharks in the Indian Ocean (Davies et al., 2019).

#### 4.6.2 Populations and spawning areas

The relationship between these genetic populations and spawning areas is a clear priority for management and conservation. The spawning areas for the North and South Pacific are only estimated to broad regions each side of the equator (Nikolic et al., 2017; Figure 1). This raises many questions, including the following: are there multiple spawning areas within the North and South Pacific respectively, are these spawning areas static locations over the spawning season and between years, and do adults show site fidelity for spawning areas? These factors could affect the rate of gene flow between the North and South Pacific populations. Previous research has predicted that Albacore and other HMMS benefit from the protection of spawning areas (Boerder et al., 2019; Hays et al., 2019), but such measures would only be effective if Albacore spawn in consistent locations.

#### 4.6.3 Climate change and potential for adaptation

The identified putative adaptive genetic differentiation between the North and South Pacific may matter for effective fisheries management and conservation. Putatively adaptive loci have been recommended as a routine method to identify stock origin and illegal trade in Albacore fisheries (Nikolic et al., 2020). Climate change and increased sea temperatures are predicted to expand and shift the range of Albacore poleward, which is expected to reduce the relative abundance of fish toward the equator (Christian & Holmes, 2016; Dufour et al., 2010; Erauskin‐Extramiana et al., 2019; Hazen et al., 2013). Such change could reduce migration and potential for gene flow between the North and South Pacific stocks. Previous research has also predicted that the recovery of Albacore stocks in the South Pacific following further climate change will depend upon genetic adaptation (Lehodey et al., 2015). Some of the putatively adaptive loci identified in this study may be relevant for adaptation to increased temperatures or physiological stress induced by climate change (e.g., six loci aligned to heat shock protein), and therefore, the 84 sites identified in this study may be useful for future management efforts.

### 4.7 Recommendations for future genetic research on highly migratory species

In this study, we analyzed genetic variation thoroughly by iteratively testing different groupings of individuals and responding to patterns that became apparent in the data. We recommend that this approach be applied to future genetic studies of HMMS, especially for the processing of RADseq data and the detection of putatively adaptive loci. In particular, in analytical pipelines such as stacks, the identification of loci and genotypes is affected by the designation of a priori groups. Sample areas can certainly be used as such groups, but they likely do not reflect accurate populations in migratory species, especially if groups do not represent discrete spawning areas. In addition, most genome scan programs used for the detection of putatively adaptive loci rely upon measures of differentiation (e.g., *F*_ST_) among a priori groups. Collectively, this means an assumption of equivalence between sample areas and populations may bias estimates of genotypic variation and affect the detection of putatively adaptive loci. In this study, we attempted to overcome this challenge by examining numerous groupings within our data, starting from the identification of loci in stacks (Table S4). The benefit of this approach was demonstrated by the increased number of loci and putatively adaptive sites for the North and South Pacific dataset compared with the 12 sample area dataset (Table S9).

## 5 CONCLUSIONS

The results of this study indicate that Albacore in the North and South Pacific can be distinguished using 84 putatively adaptive loci, but not using the presumed neutral sites. Two individuals likely represent F1 hybrids between the North and South Pacific, and 43 Albacore potentially exhibit lower degrees of mixed ancestry. In addition, four or five migrants were potentially identified between the two populations, but none of these individuals were consistently identified across datasets. Collectively, these genetic results suggest that migration and gene flow occur across the equator, but unknown selective differences in the North and South Pacific have led to strong genetic differentiation for some parts of the Albacore genome. As with studies on other tunas (Grewe et al., 2015; Pecoraro et al., 2018) and other marine species (Longo et al., 2020; Mamoozadeh et al., 2020), RADseq and the identification of putatively adaptive loci significantly improved insight into the population structure of Albacore tuna. We recommend that future studies of Albacore population structure focus on repeated sampling and spawning areas in order to link genetic and demographic variation together. The putatively adaptive loci identified in this study should be useful for distinguishing North and South Pacific Albacore in future population genetic studies, and the potential function and adaptive significance of these sites can be investigated further once an Albacore genome is available. Lastly, future genetic studies of HMMS may benefit from an iterative approach when trying to estimate population structure.

## CONFLICT OF INTEREST

The authors have no conflicts of interest to declare.

## Supporting information

Appendix S1

Appendix S2

Appendix S3

Appendix S4

## Data Availability

Demultiplexed forward and reverse DNA sequence reads for the Albacore sequenced in this study are openly available on the NCBI sequence read archive (SRA) under: PRJNA579774, http://www.ncbi.nlm.nih.gov/bioproject/579774. Additional files are available from Dryad: https://doi.org/10.5061/dryad.6djh9w103. Files include a spreadsheet of samples and sequencing success (Appendix S1), a document containing supplementary methods, references, tables, and figures (Appendix S2), lists of identified outlier loci per genome scan program (Appendix S3), full pairwise *F*_ST_ results for the 12 sample areas (Appendix S4), and genetic data files (genepop and vcf genotype files, and fasta consensus files for outlier loci). Commands used for stacks and other software, and required input files (e.g., population maps, lists of excluded loci) are included as well, and these are also hosted on GitHub: https://github.com/fvaux/pacific_albacore_ddRADseq
