# Supplementary material for: Adaptive markers distinguish North and South Pacific Albacore amid low population differentiation
Source: Evol Appl. 2021 Feb 23;14(5):1343–64. doi: 10.1111/eva.13202 (PMC8127716; doi:10.1111/eva.13202)

APPENDIX S2: SUPPLEMENTARY MATERIAL

ADDITIONAL METHODS AND RESULTS

Detection of putatively adaptive loci in all three datasets

Putatively adaptive loci were identified by all four genome scan programs in the 12 sample area and the North and South Pacific datasets, but only fsthet identified putatively adaptive loci in the New Caledonia sex dataset (Table S9). Putatively adaptive loci were organized into a separate list if they were identified by at least three programs, and the remaining loci were categorized as presumed neutral sites. Overall, a final set of 32 and 84 putatively adaptive loci were identified for the 12 sample area and North and South Pacific datasets respectively (Table S9). All loci identified as outliers by each of the four genome scan programs for both the 12 sample area and North and South Pacific dataset are provided in Appendix S3.

Although 32 putatively adaptive loci were identified for the 12 sample area dataset, these loci appeared to reflect the distinction of the North and South Pacific populations. All but two of the 32 putatively adaptive loci for this dataset were among the 84 putatively adaptive loci identified in the North and South Pacific dataset (Table S10). As with the North and South Pacific dataset, all loci with high loadings for PC1, which separated North and South Pacific samples, were detected as putatively adaptive sites in the 12 sample area dataset. This pattern strongly indicates that the 32 putatively adaptive loci in the 12 sample area dataset reflected the distinction of North and South Pacific samples. Furthermore, this pattern for PC1 also means that the identification of putatively adaptive loci in the pcadapt genome scan program reflected the separation of the North and South Pacific in PCA, and not the distinction of any further population substructure.

As recommended to assess the accuracy of the fsthet analysis (Flanagan & Jones, 2017), comparisons of F_ST_ and observed heterozygosity (H_O_) for loci in all three datasets are shown in Figure S9.

Genetic differentiation and hybrid detection in the reference mapped 12 sample area dataset

Pairwise F_ST_ was also investigated for groups in the 12 sample area dataset using all loci. Results indicated that most sample areas could not be distinguished from one another, with low, statistically insignificant pairwise F_ST_ results after correcting for multiple tests (Table S12, Appendix S4). Twenty pairwise comparisons had low F_ST_ values but were statistically significant after multiple testing (F_ST_ range of 0.02 – 0.011; Table S12, Appendix S4). Most of these comparisons were between North and South Pacific sample areas, except for the comparison of Hawaii with Oregon and British Columbia. This pattern in F_ST_ results supports the same trend of North and South Pacific differentiation observed in the PCA results (Figure 2a). New Caledonia and the likely male-biased sample areas of Hawaii and the Philippine Sea also did not exhibit any unusual F_ST_ results compared to other areas (Table S12).

Genotypic clusters were also estimated for the 12 sample area dataset in structure. Again, two genotypic clusters were identified for putatively adaptive loci, whereas one was supported for the presumed neutral loci, and two for all loci (Figure S11). Examination of these clusters revealed the same North and South Pacific pattern and no additional groups were distinguished (Figure S12).

*De novo* assembled 12 sample area dataset

*Methods*

Since reads for Albacore were mapped to the reference genome of a different species, we also assembled the demultiplexed ddRADseq reads using the *de_novo* pipeline in stacks. Default settings were applied in *ustacks* and *cstacks* (-m 2 -M 2 -N 4 -n 1). In *ustacks*, this meant that the minimum depth of coverage used to create a stack was two (-m 2), the maximum distance (in nucleotides) allowed between stacks was two (-M 2), and the maximum distance allowed to align secondary reads to primary stacks was four (-N 4). A bounded SNP model was applied with the error rate not being allowed to exceed 5% (--bound_high 0.05). In *cstacks*, the number of mismatches allowed between sample loci when building a catalog was one (-n 1). A 12 sample area dataset (-p 12) was generated using the same filtering methods and *populations* settings listed above for the equivalent *ref_map* dataset (Table S4).

*Genetic variation*

A total of 1,171 loci were estimated for the *de novo* assembled 12 sample area dataset (Table S5). Summary statistics for the *de novo* assembled 12 sample area dataset, including expected and observed heterozygosity, F_IS_, and allelic richness, were similar to results for the reference mapped dataset (Table S8). Based on PCA, the variation exhibited in the *de novo* assembled 12 sample area dataset (1,171 loci) was similar to its reference mapped equivalent (6,446 loci), with North and South Pacific samples separated by a relatively small number of loci contributing to the first PC (1.1% of variation; Figure S8). However, the *de novo* dataset exhibited greater overlap between these groups and the three clusters observed in the reference mapped dataset were not apparent. This difference may be due to information provided by the additional 5,275 loci in the reference mapped dataset (Tables 2 and A5). Given the overall similarity of these datasets, the *de novo* 12 sample area dataset was not explored any further.

SUPPLEMENTARY REFERENCES

Chow S., Ushiama H. (1995). Global population structure of albacore (*Thunnus alalunga*) inferred by RFLP analysis of the mitochondrial ATPase gene. *Marine Biology*, 123, 39–45.

Graves J.E., Dizon A.E. (1989). Mitochondrial DNA sequence similarity of Atlantic and Pacific albacore tuna (*Thunnus alalunga*). *Canadian Journal of Fisheries and Aquatic Sciences*, 46(5), 870–873.

Pujolar, J.M., Roldán, M.I., Pla, C. (2003). Genetic analysis of tuna populations, *Thunnus thynnus thynnus* and *T. alalunga. Marine Biology*, 143, 613–621.

Takagi, M., Okamura, T., Chow, S., Taniguchi, N. (2001). Preliminary study of albacore (*Thunnus alalunga*) stock differentiation inferred from microsatellite DNA analysis. *Fishery Bulletin*, 99(4), 697–701.

Viñas J., Santiago J., Pla C. (1999). Genetic characterisation and Atlantic-Mediterranean stock structure of albacore, *Thunnus alalunga. Collective Volume of Scientific Papers ICCAT,* 49, 188–191.

Yeh S.Y., Hui C.F., Treng T.D., Kuo C.L. (1996). Indian Ocean albacore stock structure studies by morphometric and DNA sequence methods*. IOTC Proceedings, 6^th^ Expert Consultation on Indian Ocean Tunas,* 258–263.

SUPPLEMENTARY TABLE LEGENDS

TABLE S1

A table summarizing previous genetic research on Albacore, listing the type of genetic marker used, number of loci, and sampling across different oceanic regions. Papers cited directly in the main text are referenced there, and any remaining citations in this table are referenced in this appendix.

Footnotes:
* This study used an RFLP assay and did not sequence DNA.

† Some of these samples were collected close to the middle of these regions and may be binned differently outside of this table (e.g. in this study, samples from the NEP and Hawaii are labelled separately in Table 1 and Figure 1).

TABLE S2

Sequencing results for the four sequencing runs (91 or 92 unique individuals per plate) on the Illumina Hi-Seq 3000 platform. The table lists the number of reads removed during quality control using the *process_radtags* component of Stacks 2.41. The percentage of PhiX reads estimated by the Illumina platform is also provided, along with total tallies and percentages before and after filtering.

TABLE S3

The mean percentage of properly paired reads successfully mapped to each tuna reference genome, based on the flagstat command in samtools 1.4. Mapping success is provided for each sample area and for all 308 individuals per reference genome. The reads used were for the 308 Albacore individuals used in the main analysis. Reads were mapped using the BWA-MEM algorithm in BWA 0.7.12.

TABLE S4

Results of varying certain parameters in *ustacks*, *cstacks* and *populations* components of Stacks 2.41. Complete lists of parameters used for each component are provided in the Methods of the main text. The mean heterozygote miscall rate estimated by the R package whoa is provided for each Stacks run. Putative paralogous sequence variants (PSVs) and loci in linkage disequilibrium (LD) were not estimated and removed for these trial runs. Rows highlighted in yellow were settings used for the reference mapped datasets, and the row highlighted in blue was used for the *de novo* assembled 12 sample area dataset.

TABLE S5

Settings applied in the *populations* component of stacks 2.41 for the *de novo* assembled 12 sample area dataset, and number of loci filtered and retained for analysis. The table lists how many loci were estimated and removed for being paralogous sequence variants (PSVs), being out of linkage disequilibrium (LD), for having low coverage depth, or for being coverage depth outliers. No loci were identified to be out of Hardy-Weinberg proportions (HWP). The last column provides the estimated heterozygote miscall rate for each dataset after filtering.

TABLE S6

Mean Wang relatedness (above) and variance (below) within and among sample areas in the 12 sample area dataset (6,446 loci). Relatedness was not estimated for Baja California as the sample size was too small (n = 4).

TABLE S7

Mean Wang relatedness (above) and variance (below) within and among the North and South Pacific populations in the North and South Pacific dataset (12,872 loci).

TABLE S8

Genetic summary statistics for the *de novo* assembled 12 sample area dataset. Estimated values are presented for expected heterozygosity (H_E_), the inbreeding coefficient (F_IS_), and allelic richness (AR). 95% confidence intervals are presented for F_IS_ values.

TABLE S9

Putatively adaptive loci estimated for each reference mapped dataset using the four genome scan programs: fsthet, outflank, bayescan, and pcadapt.

TABLE S10

Sharing of putatively adaptive loci between the reference mapped 12 sample area and North and South Pacific datasets.

TABLE S11

Details for the 84 putatively adaptive loci identified for the reference mapped North and South Pacific dataset.

TABLE S12

Pairwise F_ST_ values for all loci in the reference mapped 12 sample area dataset. Values listed in bold were statistically significant after the correction for multiple testing (provided in Appendix S4). 95% confidence intervals are not provided here, but are presented within a full spreadsheet of results (Appendix S4).

TABLE S13

A table listing all individuals identified by geneclass2 as F1 hybrids. The ‘Pacific Ocean

population’ column indicates the origin of the sample within the Pacific Ocean. The ‘Probability’ column provides the p-value for identifying an individual as an F1 hybrid, which used a critical threshold of 0.01. The North and South ‘negLog(L)’ columns provide the likelihood values for assigning an individual to the North or South Pacific populations. The last two columns show the placement of individuals among the three clusters identified along PC1 in the 12 sample area and North and South Pacific datasets; samples within Cluster B were expected to be identified as F1 hybrids.

TABLE S14

Alignment results using NCBI blastn for the 74 Pacific Bluefin genome contigs containing the 84 putatively adaptive loci in the reference mapped North and South Pacific dataset. A total of 65 of the 74 Pacific Bluefin genome contigs successfully aligned with DNA sequences on GenBank. Eleven loci labeled with an asterisk (*) aligned directly to genes (as opposed to aligning adjacent to a gene within a contig).

TABLE S15

Potential biological functions identified by panther for 20 of the 31 unique genes that aligned Pacific Bluefin genome contigs containing putatively adaptive loci.

TABLE S16

Loci mapped to the same Pacific Bluefin genome contigs between the 84 putatively adaptive loci in the reference mapped North and South Pacific dataset, and the 89 putatively adaptive loci identified by Anderson et al. (2019).

SUPPLEMENTARY TABLES

TABLE S1

| **Paper** | **mtDNA**  [# loci] | **nDNA**  [# loci] | **North Pacific** | | **South Pacific** | | **Indian** | **North Atlantic** | | **South Atlantic** | | **Med.** | **Total**  **sampling** |
| --- | --- | --- | --- | --- | --- | --- | --- | --- | --- | --- | --- | --- | --- |
|  |  |  | *NEP* | *NWP* | *SEP* | *SWP* |  | *NEA* | *NWA* | *SEA* | *SWA* |  |  |
| Graves & Dizon, 1989 | RFLP assay* |  | 12 |  |  |  |  |  |  | 11 |  |  | 23 |
| Chow & Ushiama, 1995 | *ATPase* [1] |  |  | 10 |  |  |  | 2 |  | 1 |  |  | 13 |
| Yeh et al., 1996 | Control region [1] |  |  |  |  |  | 7 |  |  |  |  |  | 7 |
| Yeh et al., 1997 | Control region [1] |  |  |  |  |  | 5 |  |  | 5 |  |  | 10 |
| Viñas et al., 1999 | D-loop [1] |  |  |  |  |  |  | 16 |  |  |  | 8 | 24 |
| Takagi et al., 2001 |  | Microsatellites [4] |  | 1 | 1 | 1 |  | 1 |  |  | 1 |  | 5 |
| Pujolar et al., 2003 |  | Allozymes [12] |  |  |  |  |  | 31 |  |  |  | 157 | 188 |
| Viñas et al., 2004 | Control region [1] |  |  | 30† |  |  |  | 54 |  |  |  | 50 | 134 |
| Nakadate et al., 2005 | D-loop [1] | *G6PD* [1] |  |  |  |  |  | 66 |  |  | 77 | 104 | 247 |
| Wu et al., 2009 | Control region [1] |  | 30 | 145 |  |  |  |  |  |  |  |  | 175 |
| Davies et al., 2011 |  | Microsatellites [12] |  |  |  | 38 |  | 394 |  |  |  | 152 | 432 |
| Montes et al., 2012 |  | Microsatellites [12] | 90 |  | 54† | 38 | 26 | 112 |  | 83 |  | 148 | 551 |
| Albaina et al., 2013 |  | Multiplex PCR [53] | 101 |  | 55† | 30 | 24 | 109 |  | 91 |  | 50 | 460 |
| Laconcha et al., 2015 |  | Multiplex assay [75] | 83 |  |  | 84 | 136 | 523 |  | 167 | 84 | 254 | 1,331 |
| Anderson et al., 2019 |  | DArTseq [1,926 – 1,999] |  |  |  | 188† |  |  |  |  |  |  | 188 |
| Nikolic et al., 2020 |  | Microsatellites [32] |  |  |  |  | 932 |  |  |  | 947 |  | 1,879 |
| This study |  | ddRADseq [6,446 – 12,872] | 183† | 51 |  | 74 |  |  |  |  |  |  | 308 |

TABLE S2

| **Counts** | **Run 1** | **Run 2** | **Run 3** | **Run 4** | ***Totals*** |
| --- | --- | --- | --- | --- | --- |
| **Total pairs of reads** | 404,105,923 | 383,534,041 | 370,698,000 | 388,203,042 | 1,546,541,006 |
| **Total single reads**  **(R1 and R2)** | 808,211,846 | 767,068,082 | 741,396,000 | 776,406,084 | 3,093,082,012 |
| **Ambiguous barcode drops** | 46,828,508 | 41,207,502 | 80,056,188 | 33,168,970 | 201,261,168 |
| **Low quality read drops** | 435,866 | 764,302 | 303,105 | 305,198 | 1,808,471 |
| **Ambiguous RAD-tag drops** | 28,187,162 | 26,036,771 | 44,108,281 | 24,064,551 | 122,396,765 |
| **Retained pairs of reads** | 366,380,155 | 349,529,754 | 308,464,213 | 359,433,683 | 1,383,807,804 |
| **Retained single reads**  **(R1 and R2)** | 732,760,310 | 699,059,507 | 616,928,426 | 718,867,365 | 2,767,615,608 |
| **PhiX reads** | 8.1% | 7.5% | 15.7% | 5.7% | 9.3% |
| **Percentage reads filtered** | 1.3% | 1.4% | 1.1% | 1.7% | 1.4% |
| **Percentage pairs of reads retained** | 90.6% | 91.1% | 83.2% | 92.6% | 89.3% |

TABLE S3

| **RefSeq accession number** | | GCA_000418415.1 | GCA_003231725.1 | GCA_900302625.1 |
| --- | --- | --- | --- | --- |
| **NCBI assembly level** | | Contig | Scaffold | Scaffold |
| **Reference genome** | | **Pacific Bluefin** | **Atlantic Bluefin** | **Yellowfin** |
| **Sample area** | **n** | **Mean percentage of properly**  **paired reads successfully mapped** | | |
| Baja California | 4 | 96.4% | 95.1% | 82.5% |
| Southern California | 39 | 90.6% | 85.5% | 91.1% |
| Northern California | 19 | 91.7% | 86.4% | 94.1% |
| Oregon | 31 | 93.0% | 89.2% | 92.1% |
| Washington | 27 | 95.9% | 93.6% | 90.1% |
| British Columbia | 10 | 94.7% | 91.9% | 93.8% |
| Hawaii | 25 | 95.8% | 94.2% | 84.5% |
| Northwest of Hawaii | 28 | 93.9% | 89.3% | 95.1% |
| Japan | 22 | 95.9% | 93.4% | 89.2% |
| Philippine Sea | 29 | 92.1% | 87.1% | 93.0% |
| New Caledonia | 54 | 95.1% | 92.4% | 86.5% |
| Tasmania | 20 | 94.3% | 90.0% | 95.6% |
| **All** | **308** | **93.9%** | **90.2%** | **90.6%** |

TABLE S4

|  |  | ***ref map*** | ***de novo*** | |  |  | **[without filtering]** | | |
| --- | --- | --- | --- | --- | --- | --- | --- | --- | --- |
| **Label** | **# indiv.** | **Reference**  **genome** | ***ustacks*** | ***cstacks*** | **# pops** | ***populations*** | **# variant and**  **invariant loci** | **# variant**  **loci** | **Mean**  **heterozygote**  **miscall rate** |
| *De novo* all reads  12 sample areas | 308 |  | -m 3 -M 2 | -n 1 | 12 | -p 12 -r 0.9 | 2,842 | 1,200 | 3.4% |
| *De novo* unmapped reads  12 sample areas | 308 |  | -m 3 -M 2 | -n 1 | 12 | -p 12 -r 0.9 | 6 | 2 | N/A |
| *De novo* unmapped reads  12 sample areas | 308 |  | -m 3 -M 2 | -n 1 | 12 | -p 12 -r 0.5 | 214 | 157 | 15.1% |
| 12 sample areas | 308 | *T. orientalis* |  |  | 12 | -p 12 -r 0.9 | 16,836 | 6,848 | 4.6% |
| 12 sample areas | 308 | *T. orientalis* |  |  | 12 | -p 12 -r 0.85 | 16,980 | 8,102 | 5.8% |
| 12 sample areas | 308 | *T. orientalis* |  |  | 12 | -p 12 -r 0.5 | 34,446 | 22,567 | 11.3% |
| North and South Pacific | 308 | *T. orientalis* |  |  | 2 | -p 2 -r 0.95 | 33,013 | 14,362 | 4.3% |
| North and South Pacific | 308 | *T. orientalis* |  |  | 2 | -p 2 -r 0.90 | 35,904 | 19,022 | 6.3% |
| Pacific Ocean panmixia | 308 | *T. orientalis* |  |  | 1 | -p 1 -r 0.5 | 50,566 | 32,211 | 13.8% |
| North Pacific  (from full catalog) | 234  (308) | *T. orientalis* |  |  | 10 | -p 10 -r 0.9 | 16,850 | 6,921 | 4.8% |
| North Pacific | 234 | *T. orientalis* |  |  | 10 | -p 10 -r 0.9 | 16,874 | 6,952 | 4.8% |
| West Coast  (from full catalog) | 130  (308) | *T. orientalis* |  |  | 6 | -p 6 -r 0.95 | 16,545 | 4,940 | 3.7% |
| West Coast | 130 | *T. orientalis* |  |  | 6 | -p 6 -r 0.95 | 16,520 | 4,967 | 3.8% |
| New Caledonia sex  (from full catalog) | 54  (308) | *T. orientalis* |  |  | 2 | -p 2 -r 0.95 | 36,086 | 9,217 | 3.7% |
| New Caledonia sex | 54 | *T. orientalis* |  |  | 2 | -p 2 -r 0.95 | 31,190 | 9,313 | 4.3% |

TABLE S5

|  |  | **Locus representation** | |  | **Filtering** | | | | |  |  |  |
| --- | --- | --- | --- | --- | --- | --- | --- | --- | --- | --- | --- | --- |
| **n** | **Dataset** | **#**  **pops** | ***populations*** | **#**  **HWE** | **#**  **PSVs** | **#**  **LD** | **#**  **Genotype**  **depth**  **<10** | **#**  **Loci**  **depth**  **outliers** | **Total**  **excluded**  **loci** | **# final**  **variant and**  **invariant loci** | **# final**  **variant loci** | **Final mean**  **heterozygote**  **miscall rate** |
| 308 | *de novo*  12 sample areas | 12 | 12 | 0 | 16 | 1 | 0 | 12 | 29 | 2,183 | 1,171 | 3.4% |

TABLE S6

|  | **BA** | **CS** | **CN** | **OR** | **WA** | **BC** | **HW** | **HN** | **JP** | **PH** | **NC** | **TS** |
| --- | --- | --- | --- | --- | --- | --- | --- | --- | --- | --- | --- | --- |
| **BA** | 0.0848  0.0043 |  |  |  |  |  |  |  |  |  |  |  |
| **CS** | 0.0217  0.0041 | -0.0425  0.0012 |  |  |  |  |  |  |  |  |  |  |
| **CN** | 0.0226  0.0039 | -0.0449  0.0008 | -0.0471  0.0006 |  |  |  |  |  |  |  |  |  |
| **OR** | 0.0129  0.0041 | -0.0519  0.0012 | -0.0529  0.0011 | -0.0593  0.0014 |  |  |  |  |  |  |  |  |
| **WA** | 0.0168  0.0048 | -0.0503  0.0007 | -0.0516  0.0005 | -0.0585  0.0009 | -0.0580  0.0003 |  |  |  |  |  |  |  |
| **BC** | 0.0612  0.0055 | -0.0015  0.0027 | -0.0028  0.0025 | -0.0100  0.0029 | -0.0081  0.0023 | 0.0415  0.0045 |  |  |  |  |  |  |
| **HW** | 0.0626  0.0064 | -0.0057  0.0028 | -0.0075  0.0024 | -0.0144  0.0029 | -0.0127  0.0023 | 0.0373  0.0045 | 0.0358  0.0048 |  |  |  |  |  |
| **HN** | 0.0202  0.0040 | -0.0456  0.0010 | -0.0465  0.0008 | -0.0542  0.0012 | -0.0519  0.0007 | -0.0001  0.0035 | -0.0072  0.0028 | -0.0471  0.0011 |  |  |  |  |
| **JP** | 0.0142  0.0033 | -0.0525  0.0007 | -0.0536  0.0005 | -0.0600  0.0009 | -0.0593  0.0003 | -0.0103  0.0024 | -0.0146  0.0028 | -0.0532  0.0007 | -0.0601  0.0003 |  |  |  |
| **PH** | 0.0322  0.0048 | -0.0350  0.0015 | -0.0362  0.0013 | -0.0428  0.0018 | -0.0415  0.0014 | 0.0089  0.0032 | 0.0037  0.0033 | -0.0354  0.0016 | -0.0438  0.0012 | -0.0250  0.0020 |  |  |
| **NC** | 0.0281  0.0050 | -0.0327  0.0022 | -0.0356  0.0020 | -0.0408  0.0023 | -0.0398  0.0018 | 0.0081  0.0039 | 0.0039  0.0039 | -0.0344  0.0021 | -0.0397  0.0017 | -0.0229  0.0028 | -0.0016  0.0032 |  |
| **TS** | 0.0005  0.0032 | -0.0560  0.0007 | -0.0629  0.0006 | -0.0679  0.0009 | -0.0671  0.0004 | -0.0189  0.0024 | -0.0232  0.0024 | -0.0611  0.0007 | -0.0664  0.0004 | -0.0507  0.0013 | -0.0288  0.0018 | -0.0564  0.0005 |

TABLE S7

|  | **North Pacific** | **South Pacific** |
| --- | --- | --- |
| **North Pacific** | -0.03329  0.00229 |  |
| **South Pacific** | -0.03513  0.00251 | -0.01634  0.00273 |

TABLE S8

| **Map**  **label** | **Group** | **n** | **Mean**  **H_O_** | **Mean**  **H_E_** | **Mean (CI)**  **F_IS_** | **Mean**  **AR** |
| --- | --- | --- | --- | --- | --- | --- |
| ***De novo* assembled 12 sample area dataset** | | | | | | |
| BA | Baja California | 4 | 0.22 | 0.21 | -0.045  (-0.076 – -0.004) | 1.59 |
| CS | Southern California | 39 | 0.21 | 0.21 | 0.035  (0.025 – 0.045) | 1.60 |
| CN | Northern California | 19 | 0.20 | 0.21 | 0.032  (0.031 – 0.060) | 1.60 |
| OR | Oregon | 31 | 0.21 | 0.21 | 0.023  (0.013 – 0.038) | 1.60 |
| WA | Washington | 27 | 0.20 | 0.21 | 0.042  (0.042 – 0.066) | 1.59 |
| BC | British Columbia | 10 | 0.21 | 0.21 | -0.007  (-0.032 – 0.013) | 1.60 |
| HW | Hawaii | 25 | 0.20 | 0.20 | 0.014  (-0.008 – 0.028) | 1.59 |
| HN | NW of Hawaii | 28 | 0.20 | 0.21 | 0.033  (0.016 – 0.042) | 1.59 |
| JP | Japan | 22 | 0.20 | 0.21 | 0.039  (0.033 – 0.065) | 1.59 |
| PH | Philippine Sea | 29 | 0.20 | 0.21 | 0.034  (0.019 – 0.047) | 1.59 |
| NC | New Caledonia | 54 | 0.20 | 0.21 | 0.024  (0.009 – 0.028) | 1.59 |
| TS | Tasmania | 20 | 0.20 | 0.21 | 0.031  (0.021 – 0.048) | 1.59 |

TABLE S9

|  |  |  |  | **# putatively adaptive loci estimated** | | | | **Final datasets** | |
| --- | --- | --- | --- | --- | --- | --- | --- | --- | --- |
| **Dataset** | **n** | **# groups** | **total #**  **loci** | **fsthet** | **OutFLANK** | **BayeScan** | **pcadapt** | **#**  **putatively**  **adaptive** | **#**  **presumed**  **neutral** |
| 12 sample areas | 308 | 12 | 6,446 | 398 | 32 | 35 | 56 | 32 | 6,414 |
| North and South Pacific | 308 | 2 | 12,872 | 371 | 126 | 72 | 92 | 84 | 12,788 |
| New Caledonia  sex | 54 | 2 | 6,917 | 183 | 0 | 0 | 0 | 0 | 6,917 |

TABLE S10

|  |  |  | **# loci shared** | |
| --- | --- | --- | --- | --- |
| **Dataset** | **# outlier loci** | **# unique** | 12 sample areas | North and South Pacific |
| 12 sample areas | 32 | 2 |  | 30 |
| North and South Pacific | 84 | 54 | 30 |  |

TABLE S11

| **#** | **SNP**  **order** | **Pacific Bluefin contig**  **accession number** | **SNP contig**  **position** | **stacks**  **locus** | **Locus position**  **(SNP)** | **Strand** |
| --- | --- | --- | --- | --- | --- | --- |
| 1 | 291 | BADN01001553.1 | 4991 | 20427 | 30 | + |
| 2 | 294 | BADN01001564.1 | 19508 | 20527 | 55 | + |
| 3 | 296 | BADN01001570.1 | 10061 | 20599 | 69 | + |
| 4 | 297 | BADN01001572.1 | 438 | 20615 | 37 | + |
| 5 | 299 | BADN01001578.1 | 2419 | 20690 | 101 | - |
| 6 | 301 | BADN01001581.1 | 6654 | 20744 | 43 | + |
| 7 | 302 | BADN01001581.1 | 6729 | 20747 | 109 | - |
| 8 | 305 | BADN01001592.1 | 23229 | 20860 | 40 | - |
| 9 | 306 | BADN01001594.1 | 2152 | 20875 | 13 | - |
| 10 | 307 | BADN01001609.1 | 14007 | 21082 | 177 | + |
| 11 | 308 | BADN01001609.1 | 14142 | 21085 | 171 | - |
| 12 | 309 | BADN01001613.1 | 505 | 21159 | 19 | - |
| 13 | 332 | BADN01001793.1 | 365 | 23419 | 7 | + |
| 14 | 334 | BADN01001811.1 | 2299 | 23616 | 84 | - |
| 15 | 336 | BADN01001830.1 | 3328 | 23804 | 134 | + |
| 16 | 338 | BADN01001834.1 | 15149 | 23873 | 74 | - |
| 17 | 339 | BADN01001834.1 | 17712 | 23881 | 8 | - |
| 18 | 340 | BADN01001835.1 | 12128 | 23901 | 238 | + |
| 19 | 341 | BADN01001835.1 | 12194 | 23907 | 127 | - |
| 20 | 342 | BADN01001840.1 | 1656 | 23943 | 27 | + |
| 21 | 343 | BADN01001836.1 | 3257 | 23913 | 110 | - |
| 22 | 478 | BADN01002553.1 | 14639 | 32568 | 207 | - |
| 23 | 1048 | BADN01005689.1 | 4692 | 72459 | 112 | - |
| 24 | 1107 | BADN01005993.1 | 15510 | 76427 | 108 | + |
| 25 | 1108 | BADN01005993.1 | 31502 | 76445 | 126 | - |
| 26 | 1109 | BADN01005993.1 | 32895 | 76448 | 55 | + |
| 27 | 1989 | BADN01011365.1 | 5538 | 136651 | 184 | - |
| 28 | 1990 | BADN01011365.1 | 10899 | 136659 | 182 | + |
| 29 | 1991 | BADN01011378.1 | 4248 | 136762 | 42 | - |
| 30 | 1992 | BADN01011374.1 | 10251 | 136715 | 68 | - |
| 31 | 1993 | BADN01011382.1 | 4194 | 136806 | 216 | + |
| 32 | 2417 | BADN01014241.1 | 1891 | 168829 | 24 | - |
| 33 | 2418 | BADN01014245.1 | 3368 | 168842 | 25 | + |
| 34 | 2419 | BADN01014263.1 | 1269 | 168960 | 217 | - |
| 35 | 2595 | BADN01015376.1 | 1843 | 180600 | 129 | - |
| 36 | 2596 | BADN01015386.1 | 956 | 180651 | 118 | - |
| 37 | 3017 | BADN01018066.1 | 4811 | 209756 | 86 | + |
| 38 | 3018 | BADN01018066.1 | 5009 | 209758 | 65 | - |
| 39 | 3917 | BADN01024210.1 | 31269 | 273473 | 85 | - |
| 40 | 4473 | BADN01027930.1 | 9794 | 311916 | 12 | - |
| 41 | 4474 | BADN01027944.1 | 1655 | 312047 | 19 | - |
| 42 | 5810 | BADN01037973.1 | 6567 | 411695 | 257 | + |
| 43 | 5811 | BADN01037976.1 | 219 | 411712 | 27 | + |
| 44 | 5812 | BADN01037979.1 | 532 | 411755 | 34 | - |
| 45 | 6418 | BADN01042530.1 | 418 | 456853 | 23 | - |
| 46 | 6543 | BADN01043658.1 | 8812 | 467913 | 22 | - |
| 47 | 6807 | BADN01045748.1 | 2702 | 486918 | 26 | - |
| 48 | 6808 | BADN01045748.1 | 11139 | 486922 | 11 | - |
| 49 | 6809 | BADN01045748.1 | 11458 | 486925 | 170 | + |
| 50 | 6810 | BADN01045751.1 | 3996 | 486956 | 117 | + |
| 51 | 6961 | BADN01046819.1 | 1760 | 497213 | 177 | + |
| 52 | 6962 | BADN01046817.1 | 2820 | 497206 | 164 | - |
| 53 | 6963 | BADN01046821.1 | 5451 | 497233 | 39 | - |
| 54 | 7463 | BADN01050554.1 | 4410 | 530671 | 16 | - |
| 55 | 7465 | BADN01050565.1 | 4318 | 530738 | 103 | + |
| 56 | 8326 | BADN01058307.1 | 5419 | 596455 | 20 | - |
| 57 | 8327 | BADN01058315.1 | 13426 | 596492 | 32 | - |
| 58 | 8329 | BADN01058319.1 | 5341 | 596521 | 35 | + |
| 59 | 9030 | BADN01064919.1 | 8488 | 650709 | 23 | - |
| 60 | 9031 | BADN01064926.1 | 9578 | 650743 | 20 | - |
| 61 | 9651 | BADN01071321.1 | 6712 | 700010 | 73 | + |
| 62 | 9723 | BADN01072067.1 | 3785 | 705793 | 29 | - |
| 63 | 9728 | BADN01072119.1 | 2766 | 706224 | 51 | - |
| 64 | 9758 | BADN01072485.1 | 538 | 709045 | 183 | - |
| 65 | 9760 | BADN01072497.1 | 2533 | 709117 | 156 | + |
| 66 | 9912 | BADN01073936.1 | 4207 | 720069 | 166 | - |
| 67 | 10028 | BADN01075100.1 | 3259 | 728748 | 78 | + |
| 68 | 10061 | BADN01075352.1 | 4784 | 730808 | 21 | + |
| 69 | 10441 | BADN01079834.1 | 762 | 762062 | 118 | - |
| 70 | 10565 | BADN01081624.1 | 3848 | 773756 | 69 | - |
| 71 | 10585 | BADN01081993.1 | 677 | 776235 | 31 | + |
| 72 | 10864 | BADN01085047.1 | 6627 | 797025 | 81 | + |
| 73 | 11020 | BADN01086829.1 | 3408 | 808993 | 125 | + |
| 74 | 11489 | BADN01093157.1 | 8689 | 847830 | 36 | - |
| 75 | 11498 | BADN01093246.1 | 3688 | 848274 | 36 | - |
| 76 | 11616 | BADN01095261.1 | 743 | 859583 | 42 | + |
| 77 | 11617 | BADN01095263.1 | 3876 | 859594 | 18 | - |
| 78 | 11677 | BADN01096568.1 | 1079 | 866549 | 52 | - |
| 79 | 11808 | BADN01099048.1 | 7484 | 879444 | 57 | - |
| 80 | 11809 | BADN01099050.1 | 3126 | 879461 | 33 | + |
| 81 | 11939 | BADN01101214.1 | 3277 | 890951 | 237 | - |
| 82 | 12071 | BADN01103746.1 | 9135 | 903361 | 46 | + |
| 83 | 12707 | BADN01122787.1 | 352 | 978375 | 49 | + |
| 84 | 12839 | BADN01130913.1 | 1175 | 998124 | 6 | + |

TABLE S12

|  | **BA** | **CS** | **CN** | **OR** | **WA** | **BC** | **HW** | **HN** | **JP** | **PH** | **NC** | **TS** |
| --- | --- | --- | --- | --- | --- | --- | --- | --- | --- | --- | --- | --- |
| **BA** |  |  |  |  |  |  |  |  |  |  |  |  |
| **CS** | -0.0064  1.0000 |  |  |  |  |  |  |  |  |  |  |  |
| **CN** | -0.0067  1.0000 | -0.0005  0.9013 |  |  |  |  |  |  |  |  |  |  |
| **OR** | -0.0064  1.0000 | 0.0003  0.1540 | -0.0004  0.8382 |  |  |  |  |  |  |  |  |  |
| **WA** | -0.0082  1.0000 | -0.0005  0.9263 | -0.0006  0.8978 | -0.0001  0.6340 |  |  |  |  |  |  |  |  |
| **BC** | -0.0019  0.8588 | -0.0002  0.6410 | -0.0001  0.5175 | 0.0004  0.2804 | -0.0007  0.8312 |  |  |  |  |  |  |  |
| **HW** | 0.0001  0.4891 | 0.0007  0.0130 | 0.0010  0.0220 | **0.0017**  **<0.0001** | 0.0010  0.0069 | **0.0026**  **0.0006** |  |  |  |  |  |  |
| **HN** | -0.0053  0.9998 | 0.0004  0.1555 | -0.0002  0.6796 | 0.0007  0.0394 | -0.0006  0.9526 | -0.0007  0.8451 | 0.0007  0.0285 |  |  |  |  |  |
| **JP** | -0.0080  1.0000 | 0.0009  0.0097 | -0.0006  0.8963 | 0.0005  0.1108 | -0.0001  0.6060 | 0.0007  0.1800 | 0.0012  0.0039 | 0.0002  0.3306 |  |  |  |  |
| **PH** | -0.0042  0.9973 | 0.0006  0.0256 | 0.0004  0.1947 | 0.0006  0.0502 | -0.0001  0.5210 | 0.0006  0.2402 | 0.0004  0.1367 | -0.0003  0.7744 | 0.0009  0.0135 |  |  |  |
| **NC** | 0.0046  0.0221 | **0.0082**  **<0.0001** | **0.0088**  **<0.0001** | **0.0082**  **<0.0001** | **0.0079**  **<0.0001** | **0.0099**  **<0.0001** | **0.0099**  **<0.0001** | **0.0080**  **<0.0001** | **0.0071**  **<0.0001** | **0.0080**  **<0.0001** |  |  |
| **TS** | 0.0020  0.2275 | **0.0082**  **<0.0001** | **0.0090**  **<0.0001** | **0.0082**  **<0.0001** | **0.0078**  **<0.0001** | **0.0087**  **0.0001** | **0.0108**  **<0.0001** | **0.0082**  **<0.0001** | **0.0065**  **<0.0001** | **0.0087**  **<0.0001** | 0.0003  0.2301 |  |

TABLE S13

| **Sample** | **Pacific Ocean**  **population** | **Probability** | **North**  **negLog(L)** | **South**  **negLog(L)** | **12 sample area**  **PC1 cluster** | **North and South**  **Pacific**  **PC1 cluster** | **Intermediate structure assignment? (0.4 – 0.6)** |
| --- | --- | --- | --- | --- | --- | --- | --- |
| OR23075 | North | 0.0020 | 41.874 | 34.905 | C | A | Y |
| HN12059 | North | <0.0001 | 47.431 | 25.925 | C | A |  |
| JP88807 | North | 0.0020 | 40.294 | 33.206 | C | A | Y |
| PH12027 | North | <0.0001 | 49.984 | 19.289 | C | A |  |
| JP88811 | North | <0.0001 | 45.317 | 21.038 | C | B |  |
| PH11948 | North | 0.0010 | 41.143 | 32.962 | B | B | Y |
| NC00016 | South | <0.0001 | 23.136 | 45.859 | B | B |  |
| NC00041 | South | <0.0001 | 27.942 | 60.29 | B | B |  |
| NC00046 | South | 0.0040 | 26.863 | 29.252 | B | B | Y |

TABLE S14

| **Loci** | **Pacific Bluefin**  **contig** | **Best BLAST**  **match** | **E value** | **Organism** | **Annotation** | **Human**  **Entrez ID** |
| --- | --- | --- | --- | --- | --- | --- |
| 20427 | BADN01001553.1 | XM_023269533.1 | 3.00E-59 | *Amphiprion ocellaris* | double C2-like domain-containing protein beta | 8447 |
| 20527 | BADN01001564.1 | HQ021522.1 | 5.36E-21 | *Sparus aurata* | Unannotated genome sequence |  |
| 20599 | BADN01001570.1 | XM_004551165.1 | 1.58E-79 | *Maylandia zebra* | transmembrane protein 248 |  |
| 20615 | BADN01001572.1 | JQ780820.1 | 7.34E-78 | *Oplegnathus fasciatus* | akirin 2 gene | 55122 |
| 20690 | BADN01001578.1 | XM_005916014.1 | 8.77E-63 | *Haplochromis burtoni* | transmembrane protein 132E | 124842 |
| 20744*, 20747* | BADN01001581.1 | XM_005458245.1 | 3.01E-82 | *Oreochromis niloticus* | transmembrane protein 132E | 124842 |
| 20860 | BADN01001592.1 | JX838795.1 | 2.09E-135 | *Oplegnathus fasciatus* | interferon regulatory factor 5 | 3663 |
| 20875 | BADN01001594.1 | AB438031.1 | 7.41E-105 | *Thunnus orientalis* | heat shock protein beta-1 | 3315 |
| 21082, 21085 | BADN01001609.1 | FQ310506.3 | 4.53E-34 | *Dicentrarchus labrax* | Unannotated genome sequence |  |
| 21159 | BADN01001613.1 | AC146541.2 | 2.21E-38 | *Gasterosteus aculeatus* | Unannotated genome sequence |  |
| 23419 | BADN01001793.1 | XM_006785214.1 | 3.47E-89 | *Neolamprologus brichardi* | protein FAM57B-like | 83723 |
| 23616 | BADN01001811.1 | FQ310508.3 | 2.98E-25 | *Dicentrarchus labrax* | Unannotated genome sequence |  |
| 23873, 23881 | BADN01001834.1 | FQ310507.3 | 2.99E-14 | *Dicentrarchus labrax* | Unannotated genome sequence |  |
| 23901, 23907 | BADN01001835.1 | FQ310508.3 | 9.73E-27 | *Dicentrarchus labrax* | Unannotated genome sequence |  |
| 23943 | BADN01001840.1 | HQ447060.1 | 1.75E-31 | *Dissostichus mawsoni* | antifreeze glycoprotein/trypsinogen-like protease | 5644 |
| 32568 | BADN01002553.1 | EF095369.1 | 1.06E-21 | *Maccullochella peelii peelii* | microsatellite Mpe3.B01 |  |
| 72459 | BADN01005689.1 | XM_005933564.1 | 1.21E-82 | *Haplochromis burtoni* | NT-3 growth factor receptor-like | 4908 |
| 76427, 76445, 76448 | BADN01005993.1 | AB438031.1 | 3.80E-129 | *Thunnus orientalis* | heat shock protein beta-1 | 3315 |
| 136651, 136659 | BADN01011365.1 | CT956064.14 | 2.84E-13 | *Danio rerio* | Unannotated genome sequence |  |
| 136715 | BADN01011374.1 | JQ723587.1 | 4.14E-79 | *Siniperca chuatsi* | microsatellite CD77 |  |
| 136762* | BADN01011378.1 | XM_005916093.2 | 2.15E-179 | *Haplochromis burtoni* | neural cell adhesion molecule 1-like | 4684 |
| 136806 | BADN01011382.1 | DQ889024.1 | 3.60E-86 | *Paralichthys olivaceus* | microsatellite Poli1460TUF |  |
| 168829 | BADN01014241.1 | FQ310506.3 | 8.54E-26 | *Dicentrarchus labrax* | Unannotated genome sequence |  |
| 168842 | BADN01014245.1 | FQ310508.3 | 1.65E-22 | *Dicentrarchus labrax* | Unannotated genome sequence |  |
| 168960* | BADN01014263.1 | XM_006785138.1 | 4.09E-75 | *Neolamprologus brichardi* | brichardi histone deacetylase complex subunit | 3065 |
| 180651 | BADN01015386.1 | JN746059.1 | 1.43E-24 | *Cyprinus carpio* | microsatellite clone 504805 |  |
| 209756*, 209758 | BADN01018066.1 | XM_003453713.2 | 1.29E-53 | *Neolamprologus brichardi* | ELMO domain-containing protein 1-like | 55531 |
| 273473* | BADN01024210.1 | XM_006785073.1 | <1.00E-200 | *Neolamprologus brichardi* | G protein-activated inward rectifier potassium channel 4-like | 3761 |
| 311916 | BADN01027930.1 | CR848746.23 | <1.00E-200 | *Danio rerio* | Unannotated genome sequence |  |
| 312047 | BADN01027944.1 | XM_003453956.2 | 1.17E-77 | *Oreochromis niloticus* | CCR4-NOT transcription complex subunit 8 | 9337 |
| 411695 | BADN01037973.1 | FQ310506.3 | 1.72E-21 | *Dicentrarchus labrax* | Unannotated genome sequence |  |
| 411755 | BADN01037979.1 | FQ310506.3 | 4.22E-68 | *Dicentrarchus labrax* | Unannotated genome sequence |  |
| 456853 | BADN01042530.1 | XM_005463809.1 | 1.23E-97 | *Oreochromis niloticus* | glutamate receptor 1-like | 2890 |
| 467913 | BADN01043658.1 | AL844187.13 | 2.61E-22 | *Danio rerio* | Unannotated genome sequence |  |
| 486918, 486922, 486925 | BADN01045748.1 | FQ310506.3 | 1.29E-36 | *Dicentrarchus labrax* | Unannotated genome sequence |  |
| 486956 | BADN01045751.1 | JQ710660.1 | 2.45E-20 | *Oplegnathus fasciatus* | Complement component C8 alpha chain | 731 |
| 497206 | BADN01046817.1 | XM_005927872.1 | 1.39E-61 | *Haplochromis burtoni* | polypeptide N-acetylgalactosaminyltransferase 10-like | 55568 |
| 497213 | BADN01046819.1 | FP325305.3 | 2.10E-46 | *Danio rerio* | Unannotated genome sequence |  |
| 497233 | BADN01046821.1 | XM_007573537.2 | 3.41E-49 | *Poecilia formosa* | polypeptide N-acetylgalactosaminyltransferase 10-like | 55568 |
| 530671 | BADN01050554.1 | EF441623.1 | 9.43E-123 | *Siniperca scherzeri* | growth hormone gene | 2688 |
| 530738 | BADN01050565.1 | FQ310506.3 | 2.49E-08 | *Dicentrarchus labrax* | Unannotated genome sequence |  |
| 596455* | BADN01058307.1 | XM_005458465.1 | 1.71E-78 | *Oreochromis niloticus* | ubiquitin carboxyl-terminal hydrolase 28-like | 57646 |
| 596492 | BADN01058315.1 | XM_005916097.1 | 3.91E-81 | *Haplochromis burtoni* | 5-hydroxytryptamine receptor 3A-like | 3359 |
| 596521 | BADN01058319.1 | XM_006785089.1 | 8.21E-69 | *Neolamprologus brichardi* | zinc finger and BTB domain-containing protein 16-A-like | 7704 |
| 650709 | BADN01064919.1 | FJ234939.1 | 1.81E-62 | *Scomberomorus commerson* | microsatellite Spma11 |  |
| 650743 | BADN01064926.1 | AB438031.1 | 2.01E-123 | *Thunnus orientalis* | heat shock protein beta-1 | 3315 |
| 700010* | BADN01071321.1 | BT083017.1 | 2.49E-40 | *Anoplopoma fimbria* | Cornifelin homolog B | 84518 |
| 705793* | BADN01072067.1 | XM_005915993.1 | <1.00E-200 | *Haplochromis burtoni* | brain-specific homeobox/POU domain protein 3-like | 5455 |
| 706224 | BADN01072119.1 | AY161011.1 | 1.08E-53 | *Sciaenops ocellatus* | microsatellite SOC 401 |  |
| 709045 | BADN01072485.1 | CU694464.18 | 5.30E-14 | *Danio rerio* | Unannotated genome sequence |  |
| 709117 | BADN01072497.1 | EU872185.1 | 5.61E-40 | *Paralichthys olivaceus* | phospholipase D2 | 5338 |
| 720069 | BADN01073936.1 | AC145765.2 | 1.49E-30 | *Gasterosteus aculeatus* | Unannotated genome sequence |  |
| 730808 | BADN01075352.1 | AC145765.2 | 2.98E-65 | *Gasterosteus aculeatus* | Unannotated genome sequence |  |
| 762062 | BADN01079834.1 | FQ310508.3 | 4.54E-16 | *Dicentrarchus labrax* | Unannotated genome sequence |  |
| 773756 | BADN01081624.1 | AB438031.1 | 6.88E-25 | *Thunnus orientalis* | heat shock protein beta-1 | 3315 |
| 797025 | BADN01085047.1 | XM_007561676.1 | 1.82E-132 | *Poecilia formosa* | histone H4 | 121504 |
| 847830 | BADN01093157.1 | XM_005725445.1 | 7.89E-117 | *Pundamilia nyererei* | microfibrillar-associated protein 3 | 9848 |
| 848274 | BADN01093246.1 | XM_006785148.1 | 1.13E-47 | *Neolamprologus brichardi* | hexokinase-2-like | 3099 |
| 859583* | BADN01095261.1 | XM_004551194.4 | 2.86E-44 | *Maylandia zebra* | unconventional myosin-VIIa-like | 4647 |
| 859594 | BADN01095263.1 | XM_004551194.1 | 9.61E-85 | *Maylandia zebra* | unconventional myosin-VIIa-like | 4647 |
| 866549* | BADN01096568.1 | XM_005725459.1 | 1.07E-105 | *Pundamilia nyererei* | proline rich 7 (synaptic) | 80758 |
| 879444 | BADN01099048.1 | XM_006785148.1 | <1.00E-200 | *Seriola lalandi* | claudin-14-like | 23562 |
| 879461 | BADN01099050.1 | XM_006785122.1 | 2.75E-175 | *Neolamprologus brichardi* | Krueppel-like factor 8-like | 11279 |
| 890951 | BADN01101214.1 | GU130437.1 | 7.34E-26 | *Gasterosteus aculeatus* | Unannotated genome sequence |  |
| 903361 | BADN01103746.1 | FQ310506.3 | 3.87E-15 | *Dicentrarchus labrax* | Unannotated genome sequence |  |

TABLE S15

| **Loci** | **Pacific Bluefin**  **contig** | **Human**  **Entrez ID** | **Panther GO-Slim biological process** |
| --- | --- | --- | --- |
| 20860 | BADN01001592.1 | 3663 | organic substance metabolic process |
| 23943 | BADN01001840.1 | 5644 | cellular component organization; cellular response to stimulus; establishment of localization; immune effector response; immune response; leukocyte activation; primary metabolic process; regulation of biological process; response to biotic stimulus; response to stress; signal transduction |
| 72459 | BADN01005689.1 | 4908 | catabolic process; cell communication; cell death; cellular component organization; cellular response to stimulus; execution phase of apoptosis; multicellular organism development; oxidation-reduction process; regulation of biological process; response to chemical; response to endogenous stimulus; signal transduction; system process |
| 168960 | BADN01014263.1 | 3065 | regulation of biological process |
| 209756, 209758 | BADN01018066.1 | 55531 | movement of cell or subcellular component |
| 273473 | BADN01024210.1 | 3761 | establishment of localization |
| 312047 | BADN01027944.1 | 9337 | organic substance metabolic process; regulation of biological process |
| 456853 | BADN01042530.1 | 2890 | cell communication; cellular response to stimulus; organic substance metabolic process; regulation of biological process; regulation of biological quality; signal transduction; system process |
| 530671 | BADN01050554.1 | 2688 | cellular response to stimulus; regulation of biological process; response to external stimulus; signal transduction |
| 596455 | BADN01058307.1 | 57646 | primary metabolic process |
| 596492 | BADN01058315.1 | 3359 | cell communication; cellular response to stimulus; regulation of biological quality; signal transduction; system process |
| 596521 | BADN01058319.1 | 7704 | organic substance metabolic process; regulation of biological process |
| 20875, 650743, 76427, 76445, 76448, 773756 | BADN01001594.1  BADN01005993.1  BADN01064926.1  BADN01081624.1 | 3315 | system process |
| 709117 | BADN01072497.1 | 5338 | localization of cell |
| 797025 | BADN01085047.1 | 121504 | cellular component biogenesis |
| 847830 | BADN01093157.1 | 9848 | cellular localization |
| 848274 | BADN01093246.1 | 3099 | cellular homeostasis; cellular metabolic process; organic substance metabolic process; regulation of biological quality; small molecular metabolic process |
| 859583,  859594 | BADN01095261.1  BADN01095263.1 | 4647 | anatomical structure morphogenesis; anatomical structure development; cell cycle; cellular localization; cellular response to stimulus; establishment of localization; organic substance metabolic process; regulation of biological process; regulation of biological function; signal transduction; system process |
| 879461 | BADN01099050.1 | 11279 | organic substance metabolic process |

TABLE S16

| **Pacific Bluefin**  **contig** | **North and South Pacific**  **outlier locus**  **(this study)** | **SNP contig**  **position** | **Anderson et al., 2019**  **outlier locus** | **SNP**  **contig**  **position** | **Distance between**  **SNPs (bp)** |
| --- | --- | --- | --- | --- | --- |
| BADN01001592.1 | 20860 | 23,229 | 22741470 | 22,767 | 462 |
| BADN01015386.1 | 180651 | 956 | 4618716 | 10,231 | 9,275 |
| BADN01058307.1 | 596455 | 5,419 | 4612039 | 5,452 | 33 |
| BADN01064919.1 | 650709 | 8,488 | 8107542 | 8,208 | 240 |

SUPPLEMENTARY FIGURE LEGENDS

FIGURE S1

A flow-chart summarizing the analytical pipeline used for processing, filtering and partitioning the ddRAD sequence data.

FIGURE S2

A bar graph showing coverage mean loci depth per individual in the reference mapped 12 sample area dataset (6,446 loci).

FIGURE S3

Graphs showing variation in coverage depth for loci in the reference mapped 12 sample area dataset (6,446 loci). Both plots in A) show variation in the mean coverage depth for loci, whereas both plots in B) show variation in the standard deviation of coverage depth for loci.

FIGURE S4

Plots produced using the whoa R package, used to examine the distribution of genotypes in the reference mapped datasets. The first column of figures (A, C, E) are scatterplots showing the frequency of genotypes (0 = homozygote allele A, 1 = heterozygote, 2 = homozygote allele B) in datasets. Lines drawn show the expected trend and bounds for genotypic frequencies based on Hardy-Weinberg proportions. The second column of figures (B, D, F) show the relationship between estimated heterozygote miscall rate versus mean coverage depth, with loci in each dataset organized into bins based on mean coverage depth.

1. Genotype frequencies in the 12 sample area dataset (6,446 loci).
2. Estimated heterozygote miscall rate versus mean coverage depth for the 12 sample area dataset (6,446 loci).
3. Genotype frequencies in the North and South Pacific dataset (12,872 loci).
4. Estimated heterozygote miscall rate versus mean coverage depth for the North and South Pacific dataset (12,872 loci).
5. Genotype frequencies in the New Caledonia sex dataset (6,917 loci).
6. Estimated heterozygote miscall rate versus mean coverage depth for New Caledonia sex dataset (6,917 loci).

FIGURE S5

Eigen values for all principal components estimated by principal components analysis (PCA) in the reference mapped datasets.

1. The 12 sample area dataset (6,446 loci).
2. The North and South Pacific dataset (12,872 loci).
3. The New Caledonia sex dataset (6,917 loci).

FIGURE S6

Principal components analysis (PCA) results for the reference mapped 12 sample area dataset (6,446 loci).

1. A scatterplot presenting genetic variation among all samples, with samples classified by collection year.
2. A scatterplot presenting genetic variation among all samples, with samples classified by age class (juveniles or adults).

FIGURE S7

A histogram showing membership of the three clusters (A – C) identified along the first principal component (0.8% of variation) in the principal components analysis (PCA) scatterplot of the reference mapped North and South Pacific dataset (12,788 loci).

FIGURE S8
A scatterplot presenting results for the principal components analysis (PCA) of the *de novo* assembled 12 sample area dataset (1,171 loci). Samples are classified by sample area.

FIGURE S9

Scatterplots comparing F_ST_ and expected heterozygosity (H_T_) for loci in the reference mapped datasets, produced using the R package fsthet. The top and bottom red lines show the smoothed quantiles of the dataset. Loci identified as outliers by fsthet occur in the plotted areas outside of the quantiles.

1. The 12 sample area dataset (6,446 loci).
2. The North and South Pacific dataset (12,872 loci).
3. The New Caledonia sex dataset (6,917 loci).

FIGURE S10

Mean K probability (LK) and DeltaK plots for the reference mapped North and South Pacific dataset. A and B) are plots for the 84 putatively adaptive loci, C and D) are plots for the 12,788 presumed neutral loci, and E and F) are plots for all 12,872 loci. Values of K (the true number of clusters) with the highest mean K probability and DeltaK values were chosen as the optimal number of clusters for each set of loci.

FIGURE S11

Mean K probability (LK) and DeltaK plots for the reference mapped 12 sample area dataset. A and B) are plots for the 32 putatively adaptive loci, C and D) are plots for the 6,414 presumed neutral loci, and E and F) are plots for all 6,446 loci. Values of K (the true number of clusters) with the highest mean K probability and DeltaK values were chosen as the optimal number of clusters for each set of loci.

FIGURE S12

structure bar graphs showing genotypic clusters estimated among samples in the reference mapped 12 sample area dataset. From top to bottom, bar graphs show results for the putatively adaptive loci, presumed neutral loci, and all loci. Within a graph, each vertical bar represents a separate individual and the genotypic clusters estimated among individuals are shown in different colors (light gray and charcoal). The height of a cluster within each vertical bar indicates the confidence that a particular individual is assigned to a given genotypic cluster (referred to as the membership coefficient). A bar above the three graphs denotes the sex of samples from New Caledonia, and for the remainder of samples it specifies whether sex is unknown or if the sample area (Hawaii and the Philippine Sea) is likely to be male-biased. The colored bars below the graphs indicate North or South Pacific origin, and the 12 sample areas are labelled. The dagger sign (†) indicates whether the presented clustering model was the optimal number of clusters for each set of loci.

FIGURE S13

A structure bar graph showing genotypic clusters estimated among samples in the reference mapped North and South Pacific dataset, using the 84 putatively adaptive loci. The graph is the same as Figure 5a, but 95% credibility values for cluster assignment are also illustrated using red bars for each column.

SUPPLEMENTARY FIGURES

FIGURE S1


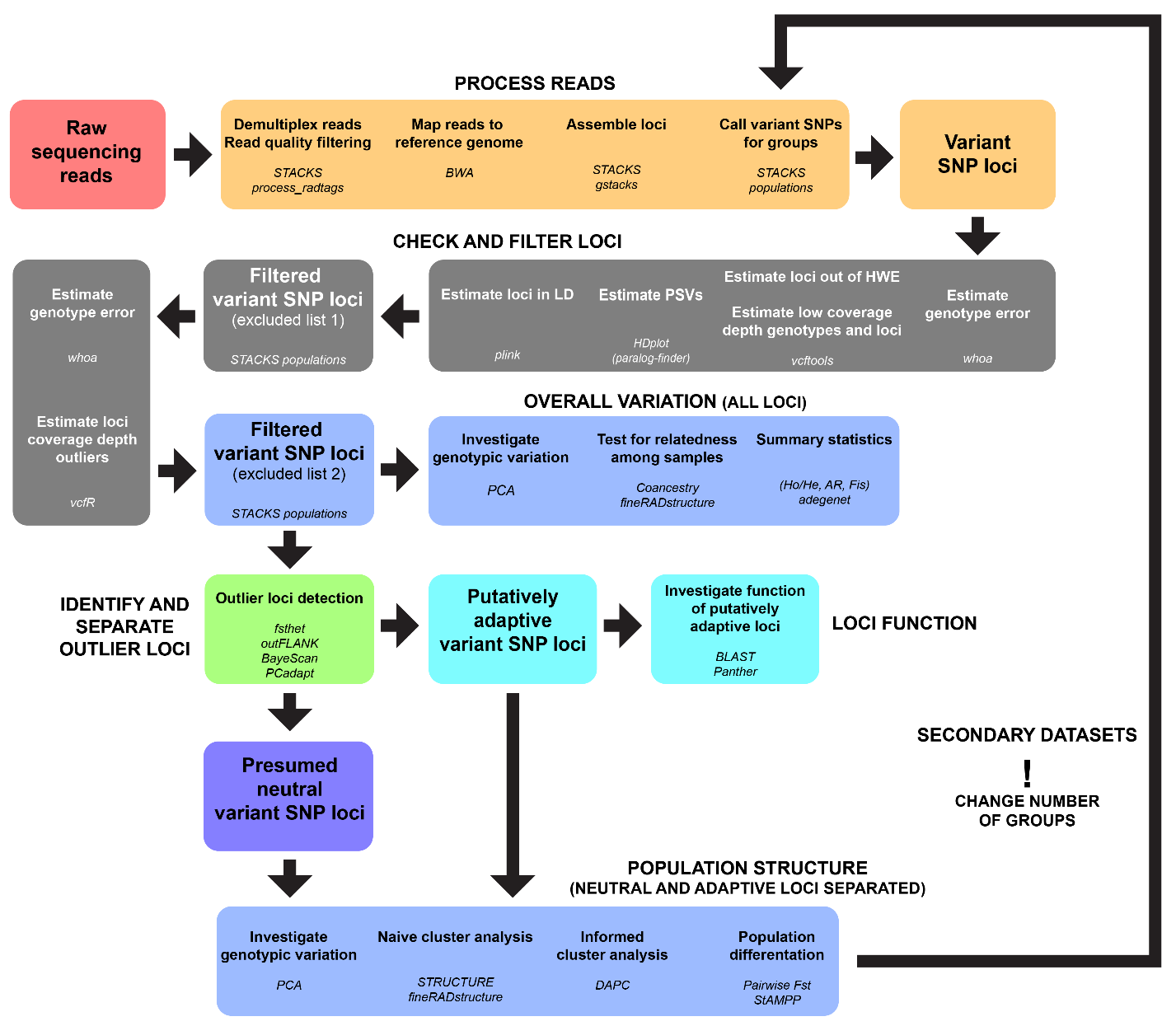


FIGURE S2


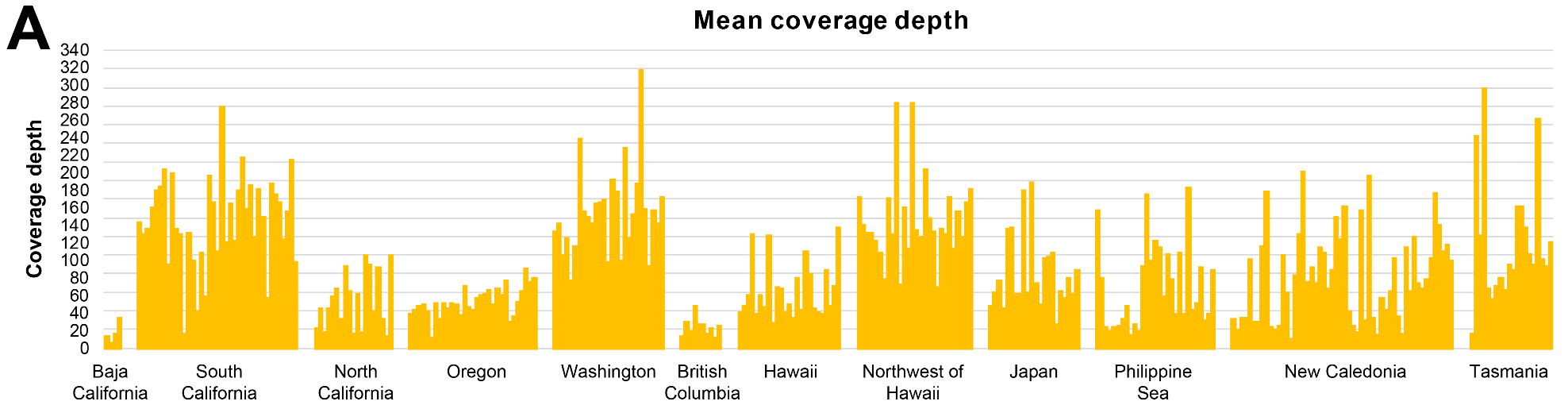


FIGURE S3


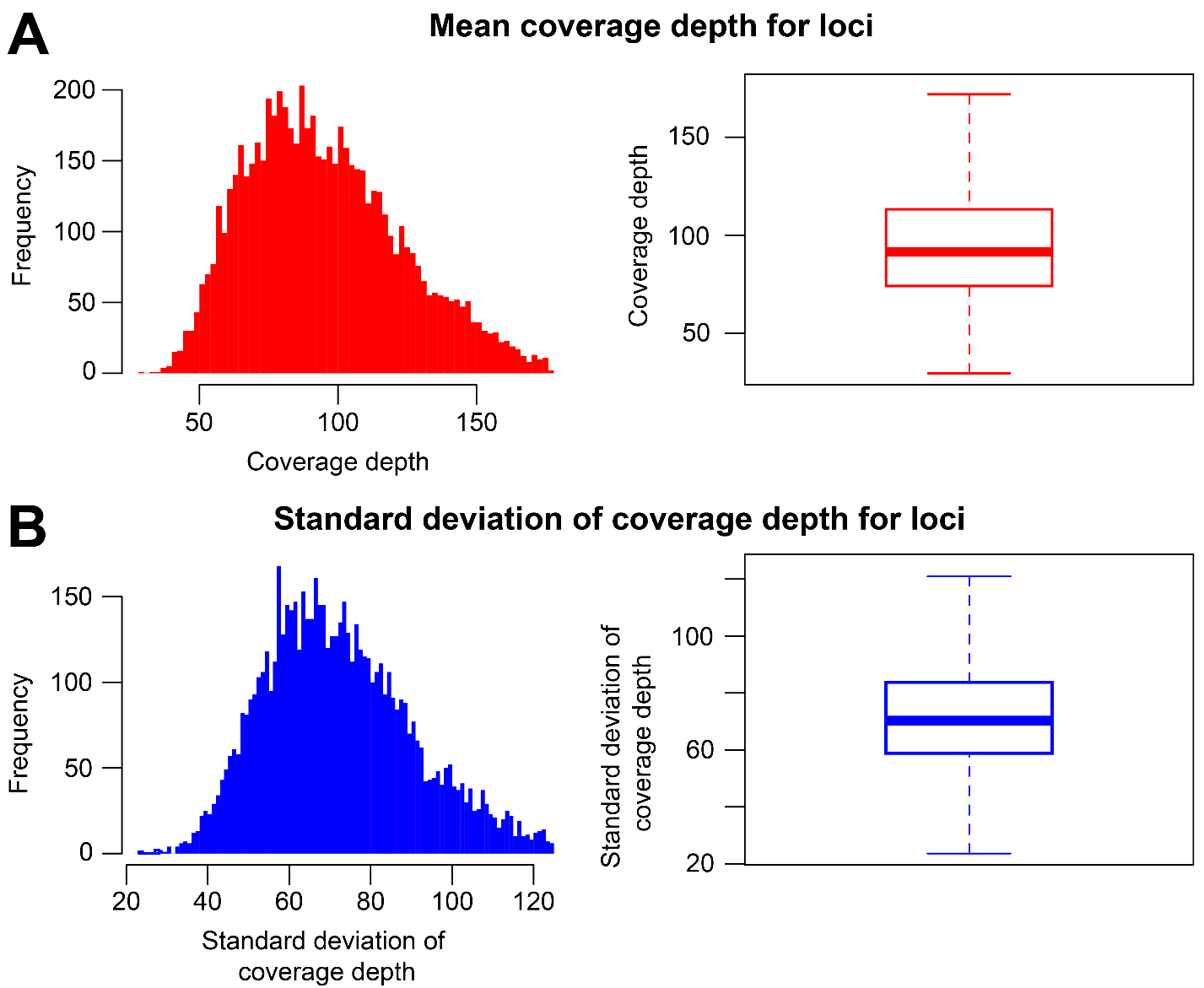


FIGURE S4


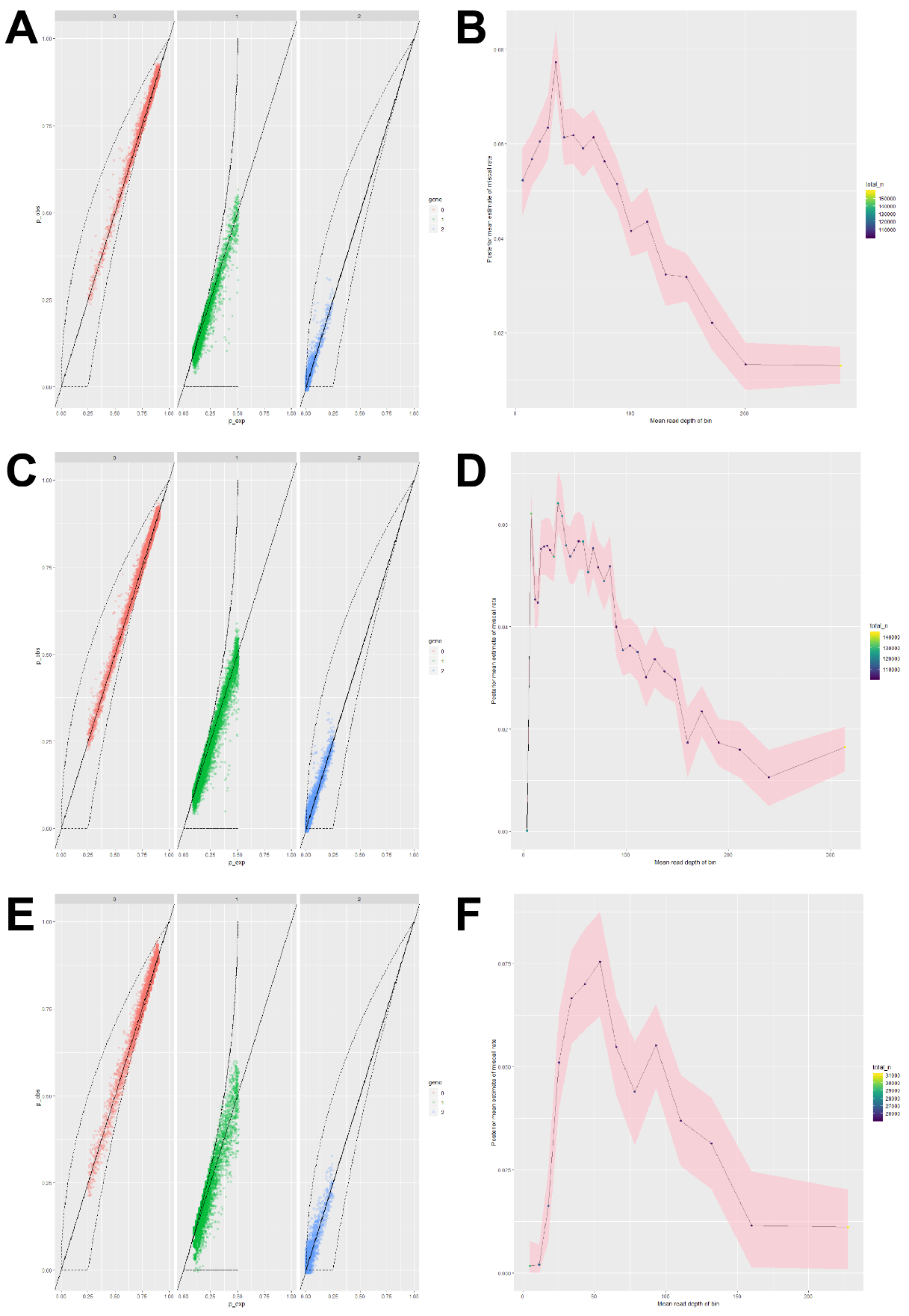


FIGURE S5


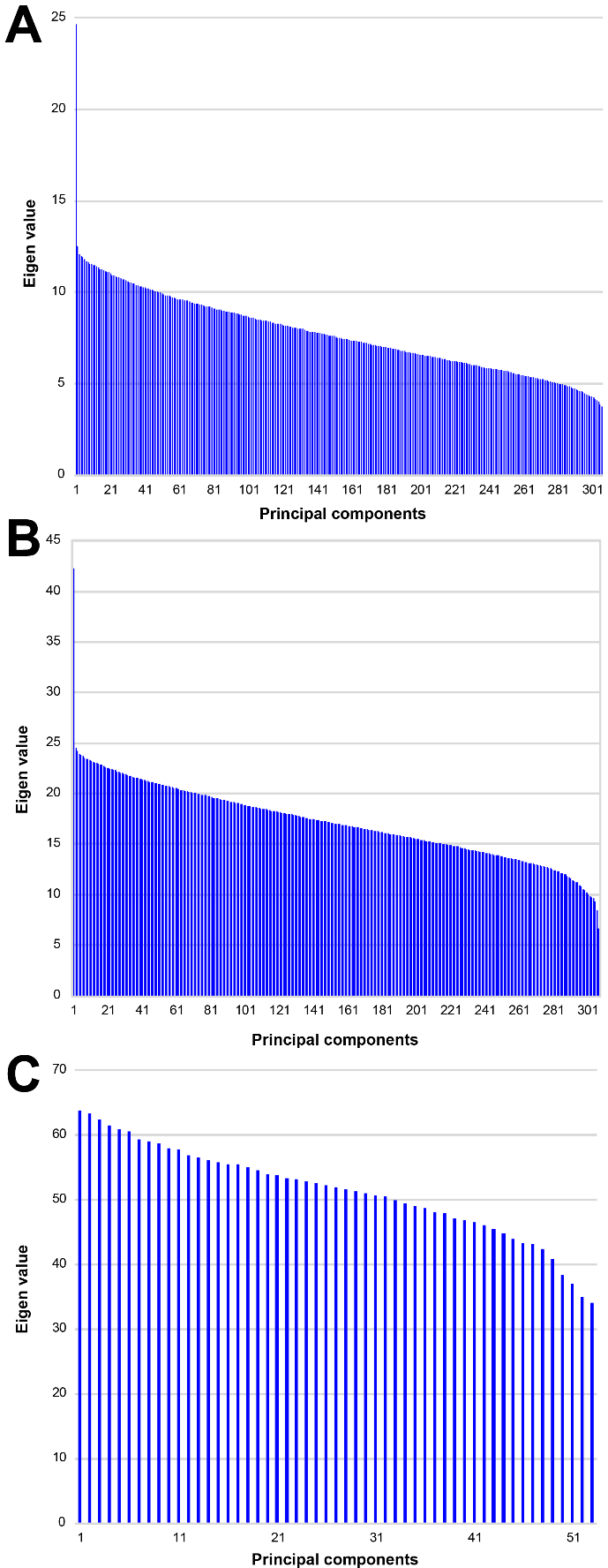


FIGURE S6


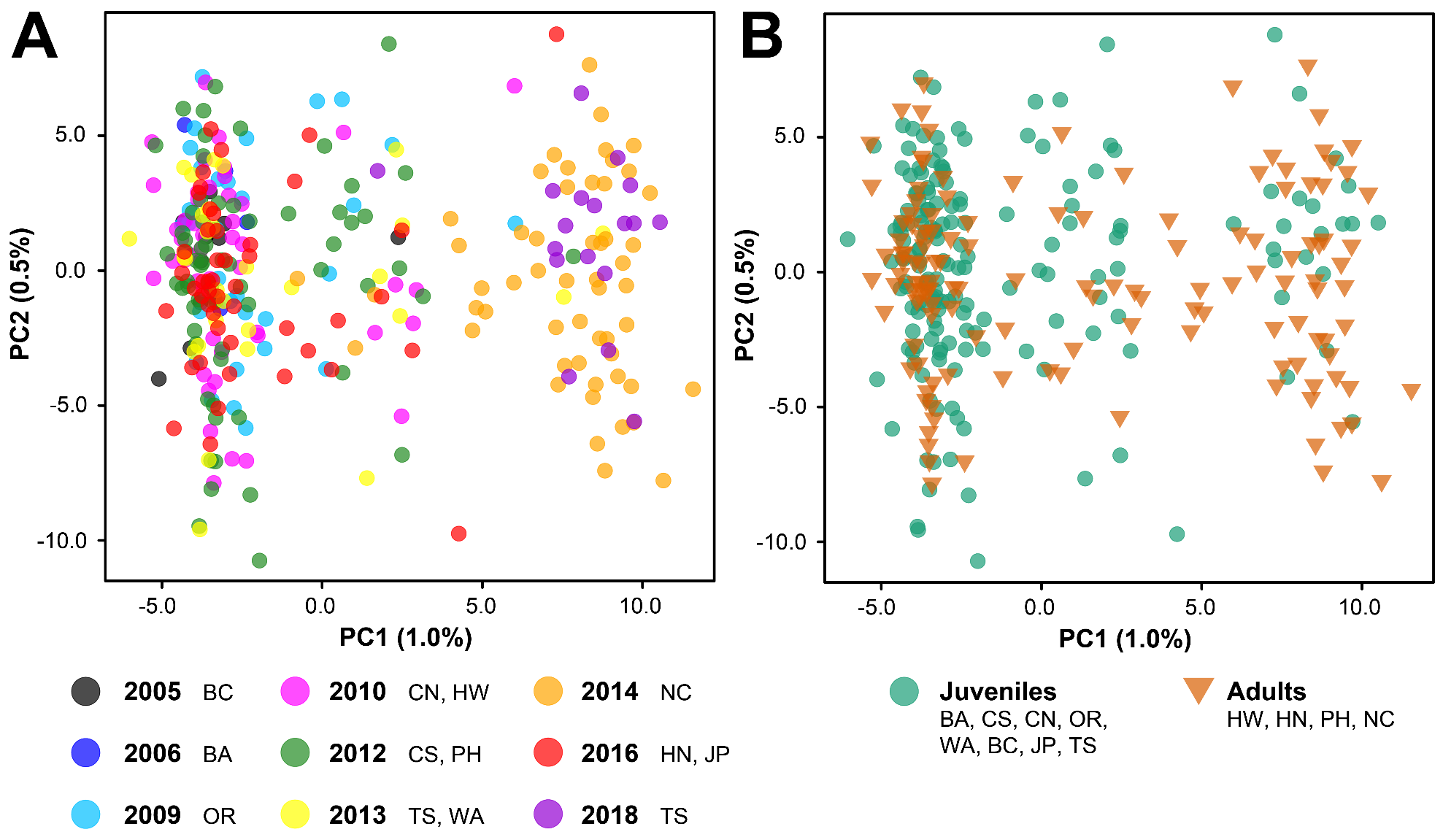


FIGURE S7


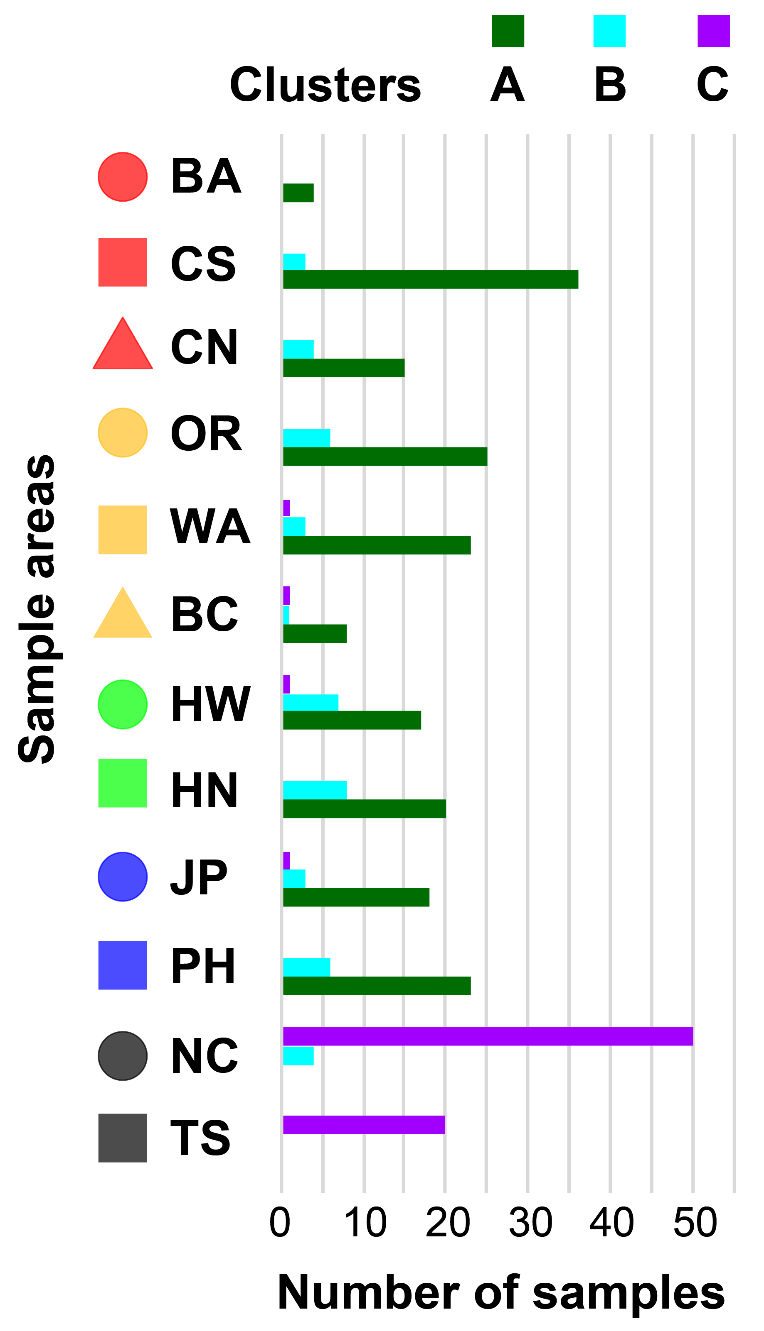


FIGURE S8


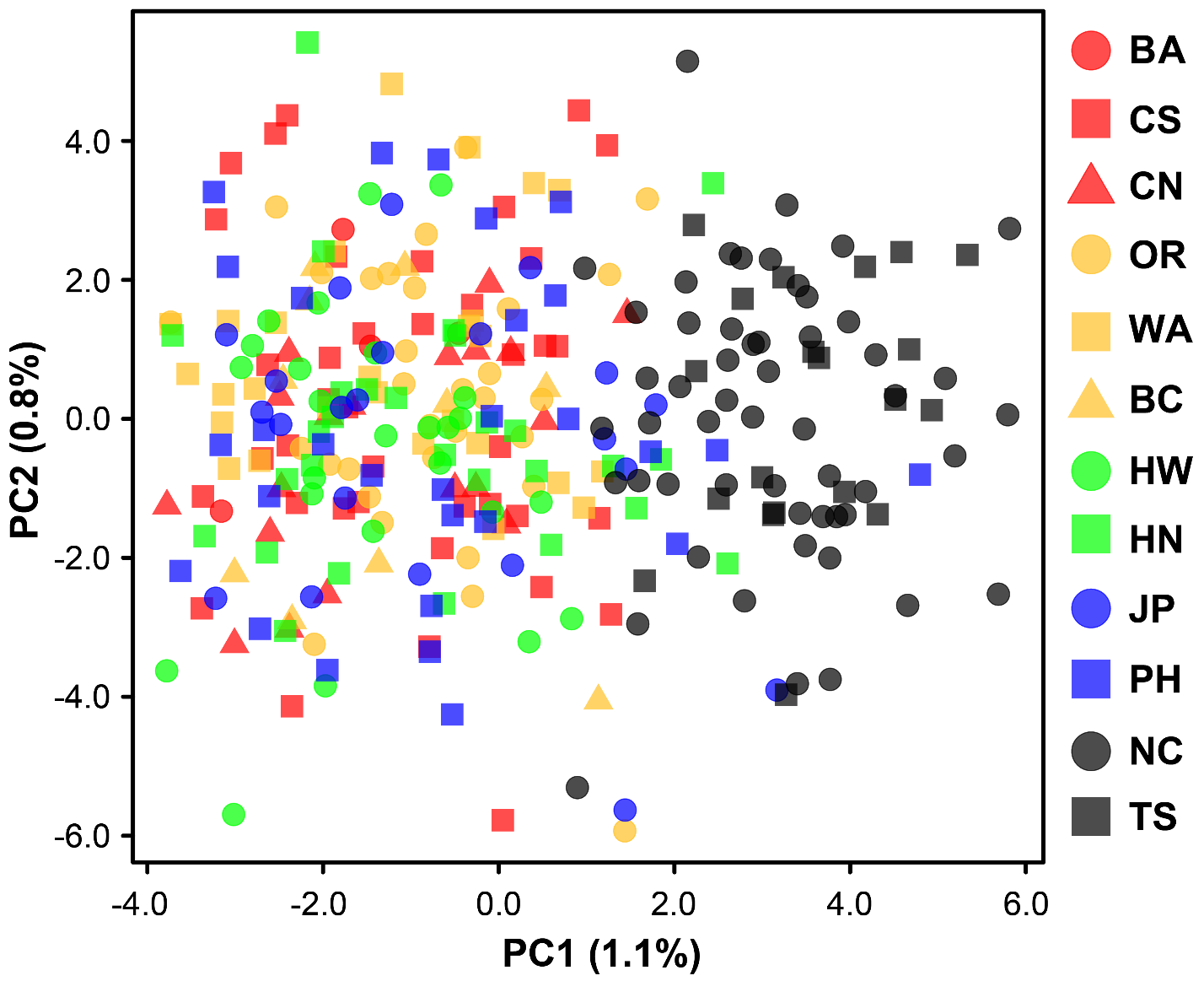


FIGURE S9


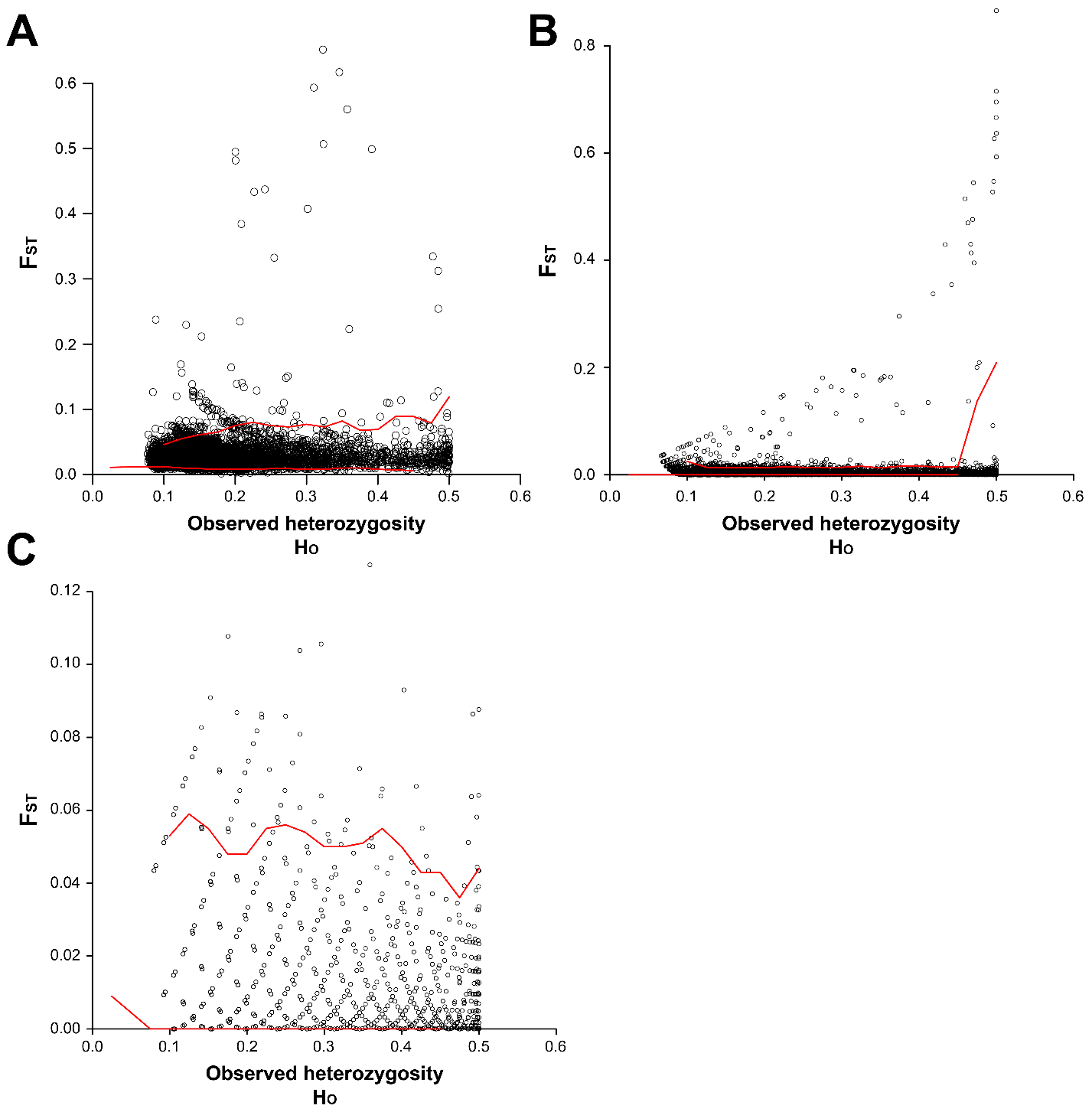


FIGURE S10


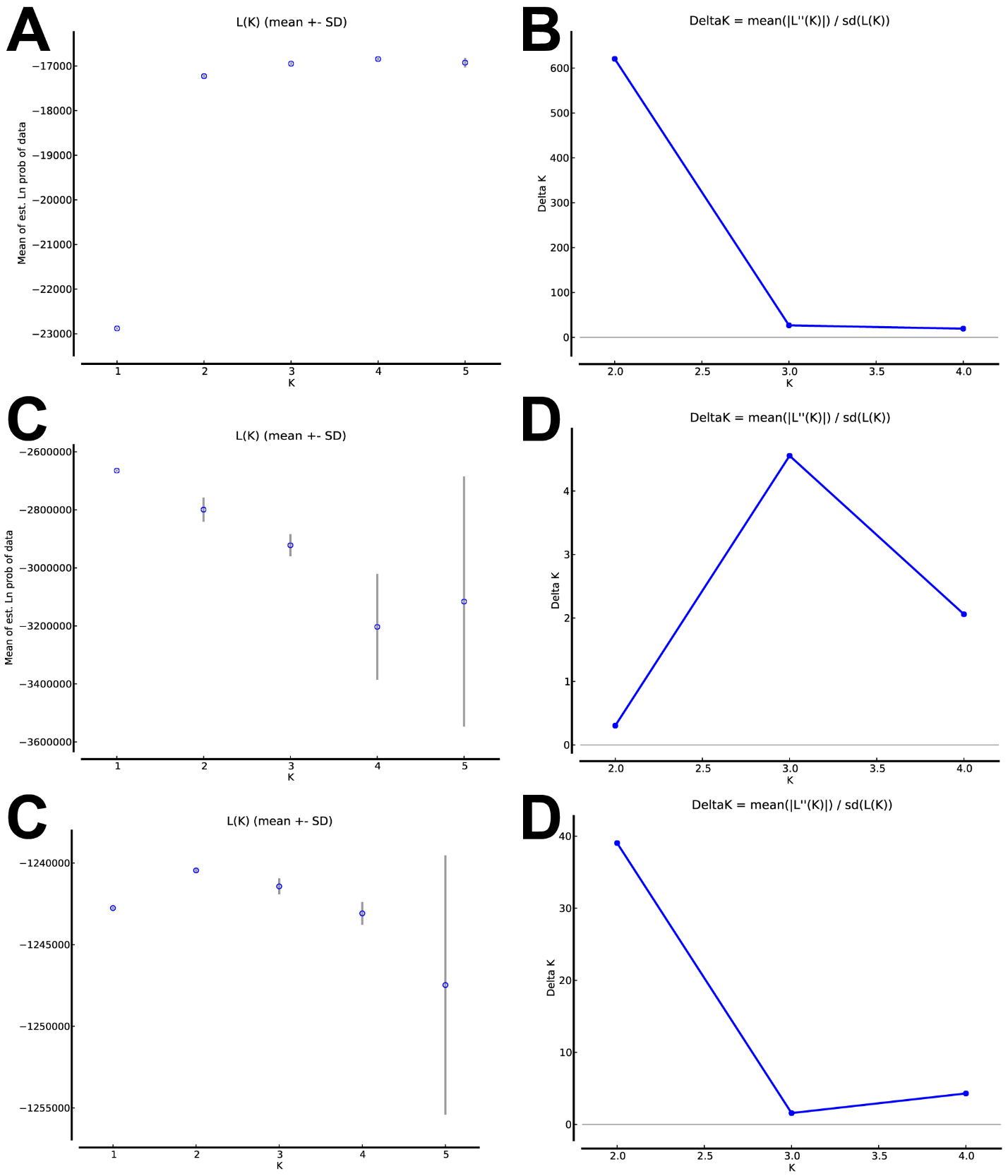


FIGURE S11


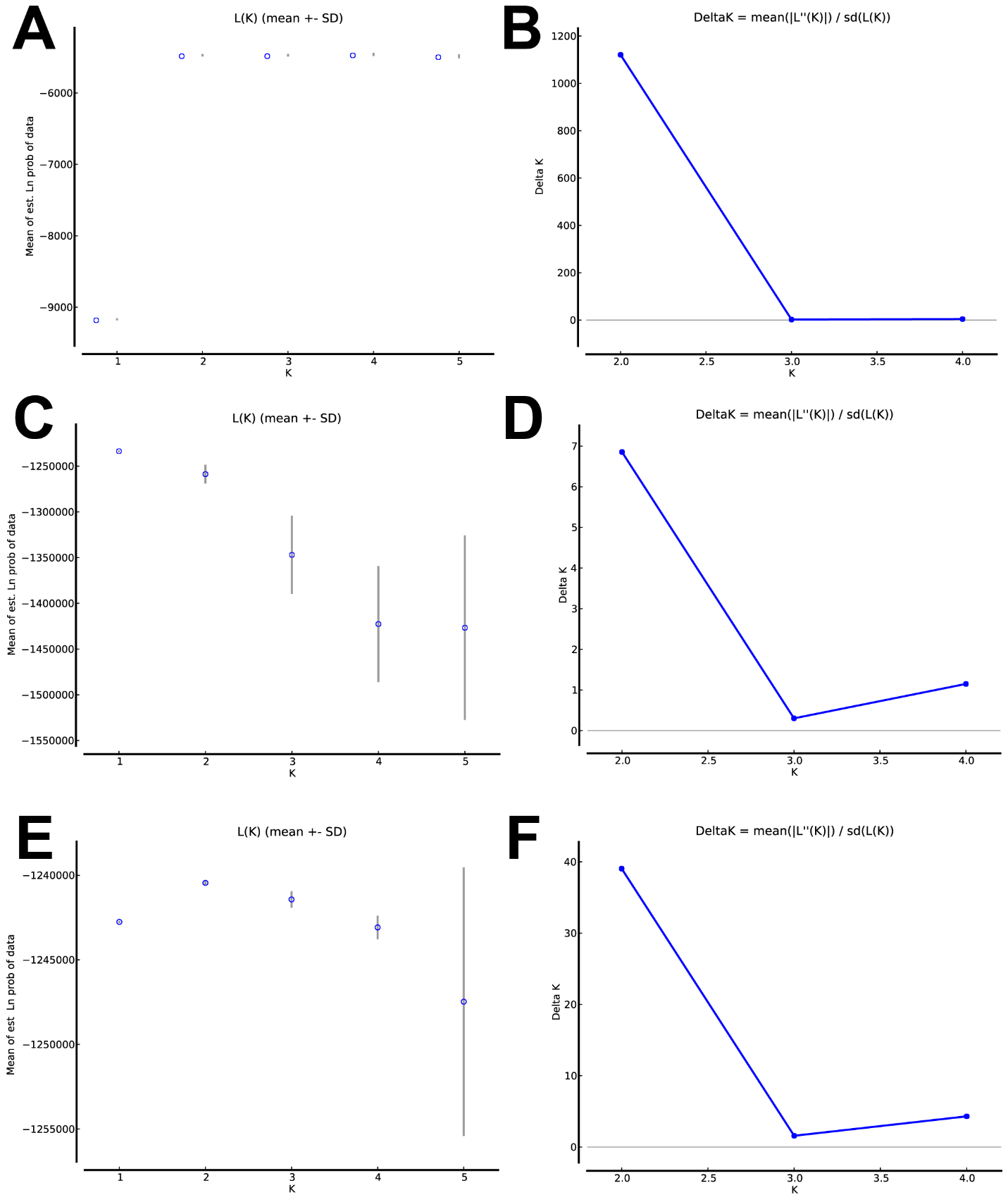


FIGURE S12


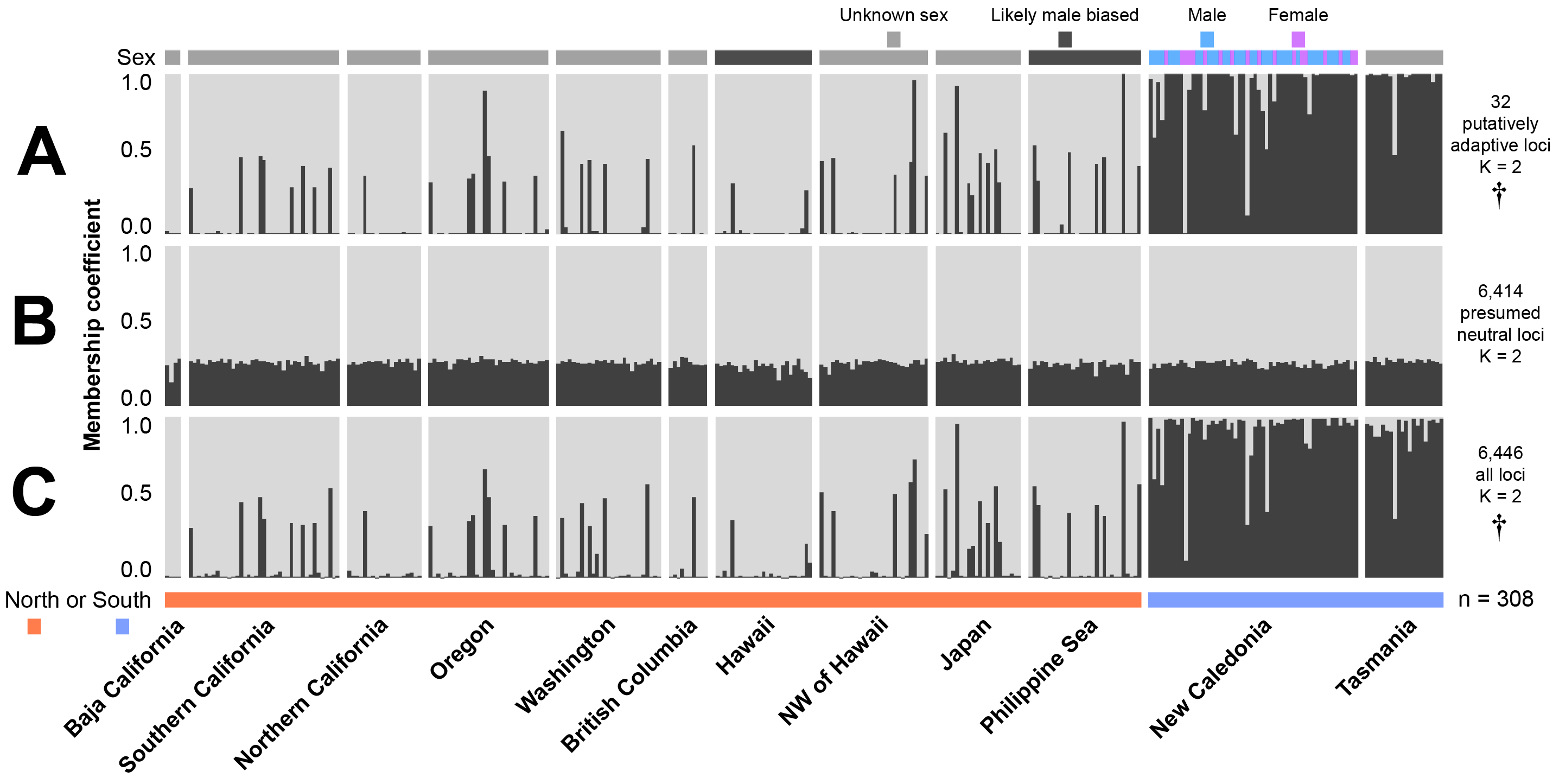


FIGURE S13


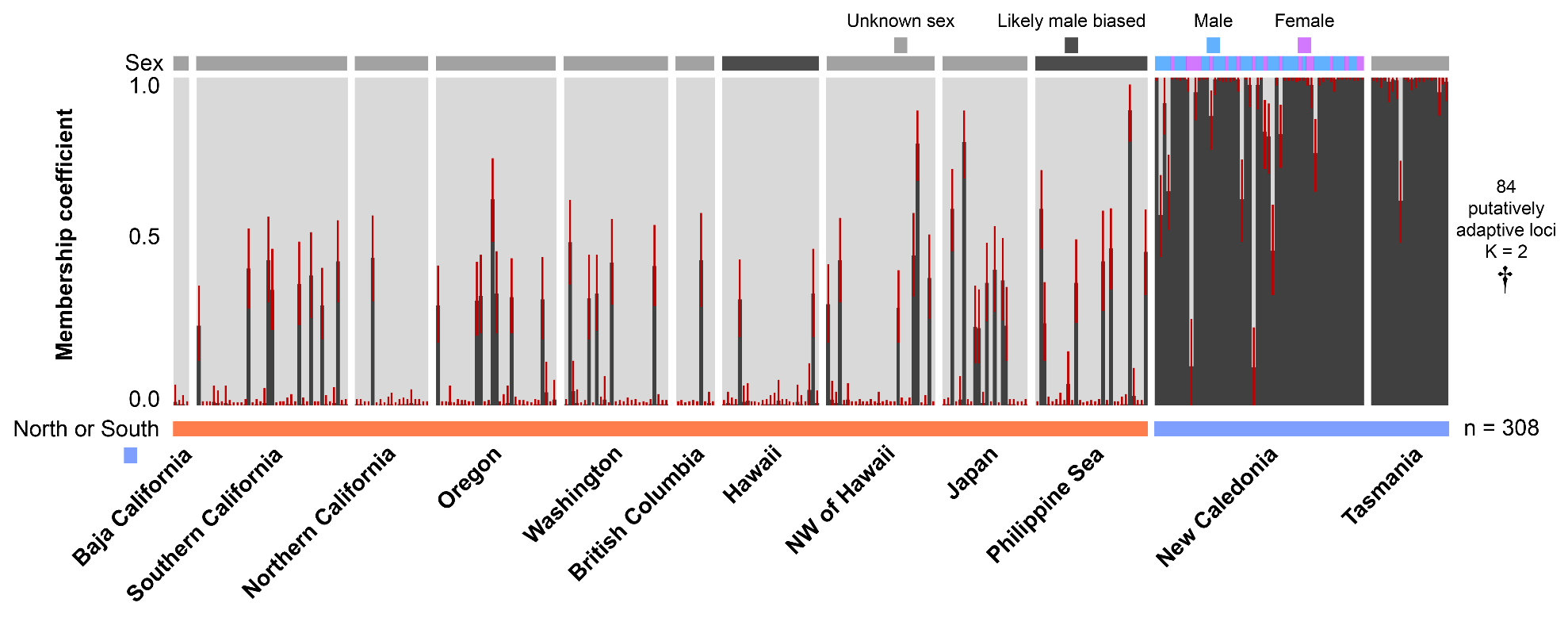

Supplement: Supplementary file 2 — Appendix S2 [file EVA-14-1343-s002.docx]
